# From panic to pedagogy: Using online active learning to promote inclusive instruction in ecology and evolutionary biology courses and beyond

**DOI:** 10.1002/ece3.6915

**Published:** 2020-10-29

**Authors:** Breanna N. Harris, Pumtiwitt C. McCarthy, April M. Wright, Heidi Schutz, Kate S. Boersma, Stephanie L. Shepherd, Lathiena A. Manning, Jessica L. Malisch, Roni M. Ellington

**Affiliations:** ^1^ Department of Biological Sciences Texas Tech University Lubbock TX USA; ^2^ Department of Chemistry Morgan State University Baltimore MD USA; ^3^ Department of Biology Southeastern Louisiana University Hammond LA USA; ^4^ Department of Biology Pacific Lutheran University Tacoma WA USA; ^5^ Department of Biology University of San Diego San Diego CA USA; ^6^ Department of Geosciences Auburn University Auburn AL USA; ^7^ Biology Department St. Mary’s College of Maryland St. Mary’s City MD USA; ^8^ Department of Advanced Studies, Leadership, and Policy Morgan State University Baltimore MD USA

**Keywords:** active learning, culturally responsive, equity, inclusive teaching, trauma‐informed, UDL

## Abstract

The rapid shift to online teaching in spring 2020 meant most of us were teaching in panic mode. As we move forward with course planning for fall and beyond, we can invest more time and energy into improving the online experience for our students. We advocate that instructors use inclusive teaching practices, specifically through active learning, in their online classes. Incorporating pedagogical practices that work to maximize active and inclusive teaching concepts will be beneficial for all students, and especially those from minoritized or underserved groups. Like many STEM fields, Ecology and Evolution shows achievement gaps and faces a leaky pipeline issue for students from groups traditionally underserved in science. Making online classes both active and inclusive will aid student learning and will also help students feel more connected to their learning, their peers, and their campus. This approach will likely help with performance, retention, and persistence of students. In this paper, we offer broadly applicable strategies and techniques that weave together active and inclusive teaching practices. We challenge instructors to commit to making small changes as a first step to more inclusive teaching in ecology and evolutionary biology courses.

## Graphical abstract

We encourage instructors to use inclusive teaching practices and active learning in their online classes. Incorporating pedagogical practices that work to maximize active and inclusive teaching concepts will be beneficial for all students, and especially those from minoritized or underserved groups. In this paper, we offer strategies and techniques that weave together active and inclusive teaching practices and challenge faculty to commit to making small changes as a first step to more inclusive teaching in ecology and evolutionary.

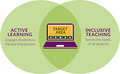

## 1 INTRODUCTION

### 1.1 Framework and challenge to instructors

In response to COVID‐19, in spring, 2020 many of us rapidly took our in‐person courses to an online format. This was panic pedagogy and we made the best of an emergency situation. Going forward, we now have a chance to reflect and think critically about how to best develop and deliver evolutionary and ecological content online. In this piece we challenge instructors to use the opportunity created by the COVID‐19 pandemic to rethink the way in which they teach. Let us leverage the situation to increase use of active and inclusive practices in our (online) classrooms. We encourage instructors to be mindful of the how and why of their course design and to embrace active and inclusive teaching practices.

**FIGURE 1:**
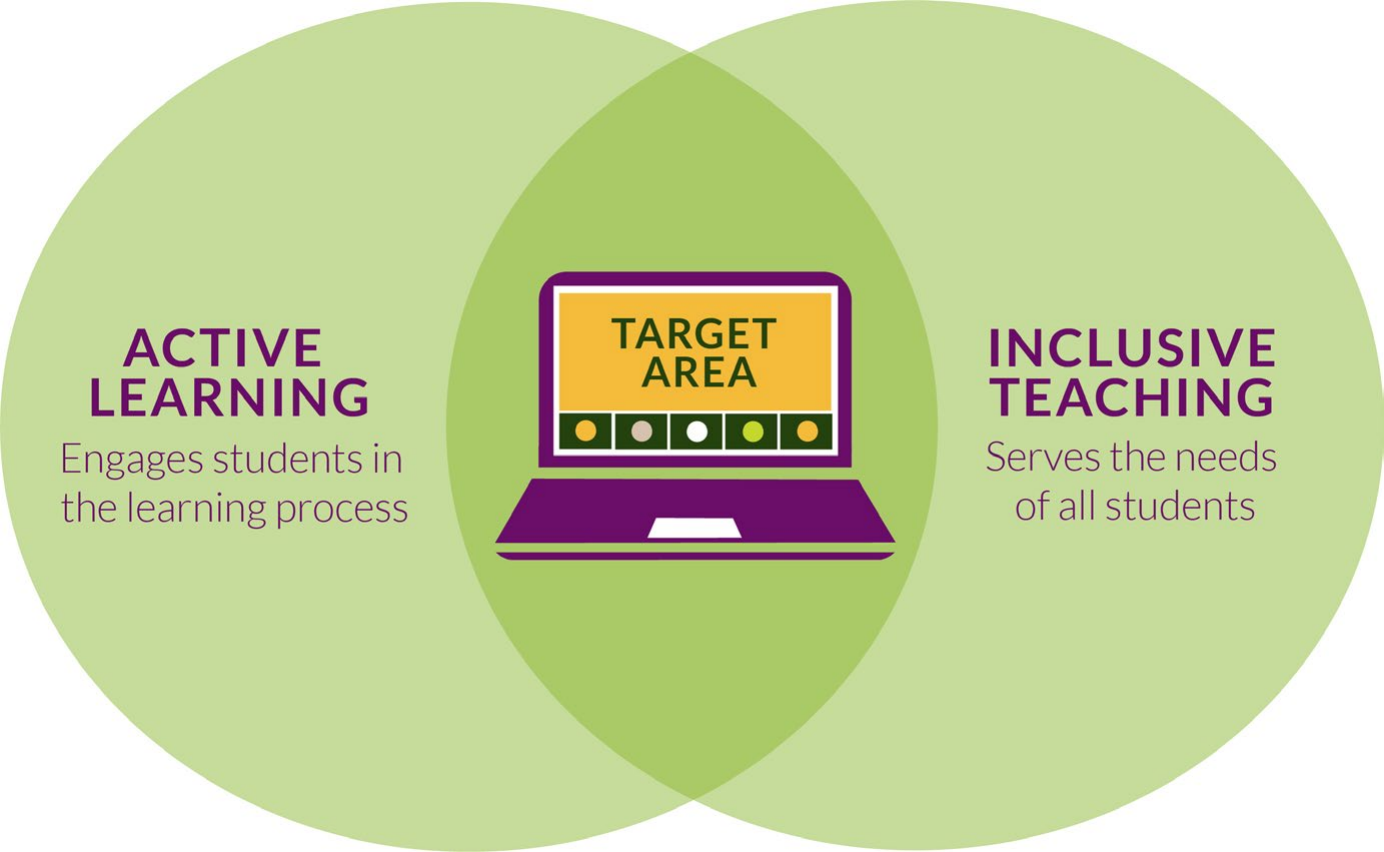
Active learning and inclusive teaching are both important pedagogical practices; however, they are not interchangeable terms. We propose instructors should aim for the area of center overlap when developing their courses

Online teaching offers opportunities to increase equity, inclusion, and overall teaching effectiveness, but courses must be intentionally designed with this outcome in mind. The foci of our manuscript are active learning and inclusive teaching in an online learning environment. Active learning is often touted as a way to maximize course effectiveness; enhance student learning; help students feel more connected to their learning, their peers, and their campus; and to increase student retention, persistence, and success. To do this, however, effective active learning practices need to be implemented in a thoughtful and inclusive way (Andrews et al., 2011; Michael, 2006). Importantly, active learning is not synonymous with inclusive teaching. Active learning is not necessarily inclusive and inclusive teaching practices are not all active in nature. We advocate that instructors design their online courses with a particular focus on the intersection between active learning and inclusive teaching (Figure 1). Classrooms and active learning exercises that embrace inclusivity provide a multi‐pronged approach to create a student‐centered learning environment that meets goals of *Vision and Change* (AAAS, 2011, 2015, 2018).

This manuscript aims to help instructors frame and define the concepts of active learning and inclusive teaching in their classrooms. Just as learning is an iterative process for students, teaching is an iterative process for educators. We would not expect students to be able to perfectly perform laboratory techniques just by reading about the methods and background. Likewise, we cannot expect instructors to master active and inclusive teaching by reading a few papers. True inclusive teaching will require continued learning, effort, commitment, and personal growth; confronting potentially uncomfortable situations; and departmental and institutional support. In this manuscript, we provide several different strategies for designing more active and inclusive online classrooms. Changing the way we teach can be difficult and overwhelming, thus we encourage readers to start small and commit to making one or two changes each semester. It is not possible, nor advised, to make all changes at one time. We hope this manuscript helps instructors progress on their journey of active and inclusive teaching. In Section 1, we provide an overview of active learning and inclusive teaching methods; in Section 2, we discuss challenges and solutions of online and active learning; and in Section 3, we provide details for three explicit active and inclusive online teaching strategies for use in ecology and evolutionary biology courses. Our learning goals for the manuscript are as follows. After reading this paper, readers will know, will have, or be able to:

1. Compare and contrast active learning and inclusive teaching. Know that these terms are not interchangeable and explain the importance of each.
2. Define and provide examples for implementation of inclusive teaching.
3. Give examples of ways to integrate inclusive teaching practices into online active learning.
4. List some common challenges and solutions associated with active learning.
5. Discuss equity concerns associated with online teaching.
6. Appreciate the difficulties of changing the way we teach and acknowledge that small pieces can be adopted over time; changes do not have to happen all at once.
7. Devise a concrete action plan to incorporate active learning and inclusive teaching practices into at least one aspect of their ecology or evolutionary biology syllabus.

### 1.2 A new way of knowing

Nearly 40 years ago biologist John Moore called for scientists in higher education to be better educators, namely to teach science as a way of knowing, focused on the processes of doing science and not on the memorization of facts (Moore, 1984). Moore's initial call spurred new directives and initiatives for biology education and for science teaching in general. In 2011, the American Association for the Advancement of Science (AAAS) published the first edition of *Vision and Change in Undergraduate Biology Education: A Call to Action*. The AAAS highlighted the need for student‐centered learning, teaching the process of science, and the integration of science with society (AAAS, 2011). *Vision and Change* also called for broadening participation and making science more inclusive. These documents have been updated over time (AAAS, 2015, 2018) but the overarching message remains the same: biology education needs to be more inclusive, to engage learners as doers, and to emphasize the interdisciplinary aspects of biology.

Along the same theme, although John Moore called for teaching science as a way of knowing, bell hooks called for a different way of knowing. She advocated for engaged pedagogy and using education as the practice of freedom (hooks, 1994). This type of teaching includes the voices and lived experiences of all students in the classroom and calls for interrogation of ideas and traditional ways of knowing (hooks, 1994). Educators should embrace cultural diversity and deconstruct academic biases that uphold racism, sexism, imperialism, and white supremacy (hooks, 1994). Instructors should also build teaching communities where students are active participants, educators embrace mind and body, and reject the “banking system of education” in favor of active engagement (hooks, 1994).

One way in which educators began to meet these calls to action is by using active learning practices that engage students in the learning process. **Active learning** requires the involvement of students in their own education and allows them to take agency over understanding and applying material. Active learning often focuses on the higher levels of Bloom's taxonomy, a common framework for understanding educational outcomes (Anderson et al., 2001; Bloom, 1956). Active learning challenges students to develop a deeper mastery of curricular knowledge by applying concepts, analyzing data, and creating novel synthesis or knowledge. Active learning methods are highly effective. As evidenced by a meta‐analysis of 225 studies, active learning practices enhance student learning and reduce failure rate in undergraduate science, technology, engineering, and math (STEM) courses (Freeman et al., 2014). Additionally, active learning approaches, accompanied by major changes to course structure, improve performance in evolution courses (Frasier & Roderick, 2011) and can be used successfully in ecology (Burrow, 2018) and in courses aiming to integrate ecological and evolutionary concepts throughout the biology curriculum (White et al., 2013).

On top of the general educational benefits associated with active learning practices, the use of these strategies in the classroom is especially beneficial for students from groups traditionally underrepresented in STEM. The Association of American Colleges and Universities (AAC&U) called for an increase in equity and availability of high‐impact educational practices for all students, but especially for underserved students, as this demographic shows the highest gains in grades and persistence when exposed to high‐impact, active learning (Kuh, 2008). Similarly, the National Institute of General Medical Sciences and the Howard Hughes Medical Institute Joint Working Group on improving persistence in STEM made evidence‐based recommendations and advocated for incorporating active learning across schools to benefit underserved students (Estrada et al., 2016). For example, when active learning practices (in this case, five major changes to the course curriculum) were used in an introductory evolutionary biology and biodiversity course, African American, Latinx, Pacific Islander, and Native American students showed higher learning assessment gains and course grades at the end of the semester compared to outcomes in the same course using traditional lecture teaching (Ballen et al., 2017). In that study, the gains were specific to students in the aforementioned minoritized groups. Learning outcome gains and grades did not differ between teaching methods for students from nonminoritized groups; however, for all students, self‐efficacy was higher in the course using active learning (Ballen, et al., 2017). In a separate study, addition of structured active learning practices (i.e., lecture‐free classes with daily clickers, hands‐on activities, and pretests) to large (>300 students) introductory biology courses increased performance of all students and was able to reduce the achievement gap between students from disadvantaged backgrounds (defined here primarily by first‐generation students and low income) and those from nondisadvantaged backgrounds (Haak et al., 2011). Lastly, a recent meta‐analysis found that active learning practices can reduce the achievement gap between students in minoritized groups (based on race, ethnicity, or income) and those in nonminoritized groups by 33% (Theobald et al., 2020).

Active learning has often been touted as a way to increase student performance as well as inclusion in the classroom, and clearly using these practices can be effective (Ballen, 2020). However, active learning is not always beneficial in helping students learn evolutionary concepts (Andrews et al., 2011) and this is likely due to the way in which instructors incorporate active learning into their courses (Michael, 2006). For example, Andrews and colleagues surveyed a random sample of introductory biology courses taught by 33 different instructors across the nation to determine whether active learning increased student understanding of natural selection (Andrews et al., 2011). The authors found that it did not. They attributed this outcome to a few possible scenarios centered on the implementation of active learning and instructor training and knowledge. Previous studies showing the effectiveness of active learning were conducted using instructors who had science education research experience. Thus, these instructors better understood nuances of active learning and were able to design effective courses and implement activities in more meaningful ways. This is not surprising given the lack of attention to learning how to teach in STEM disciplinary training (Winberg et al., 2019). Unfortunately, it seems one cannot just use clickers or periodic class discussion and expect increased gains in student learning. This is a disappointing take‐away as we all strive to be great educators and having ready‐to‐use tools to increase performance is appealing. However, this does not mean we should not try to incorporate more meaningful active learning assignments into the classroom, nor does it mean that only science education experts can effectively use active learning. What it does mean is that we all need to be more intentional about how we design and implement classroom activities. Additionally, taking the time to implement a few strategies well is better than trying to implement many strategies at once.

The rapid shift to online learning and the upending of the traditional day‐to‐day of teaching in spring, 2020 provided us all with the unique opportunity to pause and re‐calibrate our teaching. Importantly, it provided an opportunity to think critically about course design. For many of us, we are now facing the challenge of completely re‐working our course(s) for online delivery. **Scientific teaching** practices suggest that course planning should start from the learning outcomes (Handelsman et al., 2007). That is, instructors first determine what students should know and be able to do at the end of the course, and then, the course framework and assignments are designed to meet those outcomes. This approach, termed **backward design**, can facilitate creation of meaningful active learning assignments. Instructors can further increase the effectiveness of delivery by providing clear learning goals for each activity, lecture, and/or unit (Handelsman et al., 2007). These explicit, direct, and measurable goals are typically written using verbs corresponding to different areas of Bloom's Taxonomy (Crowe et al., 2008). However, to truly incorporate inclusivity in these active learning practices, we challenge instructors to consider course design above and beyond specific disciplinary content by incorporating multiple inclusive teaching strategies (see Penner, 2018).

### 1.3 Incorporating inclusive teaching practices

Foundationally, **inclusive teaching** serves the needs of all students, but no single, universally accepted definition exists (Ainscow et al., 2006; Forlin & Loreman, 2014; Miles & Singal, 2010). Rather, a spectrum of approaches to inclusive teaching exist, including teaching to accommodate various learning differences and ability levels (An & Carr, 2017; Orr & Hammig, 2009) as well as teaching that includes and empowers historically marginalized learners (Friedrich et al., 2008). Whatever the approach, the goal of inclusive teaching is to ensure that diverse learners have equal opportunities to engage in meaningful learning experiences that maximize their participation and achievement (Lawrie et al., 2017). For this manuscript, we use the broad definition proposed by Dewsbury (2017) and see inclusive teaching as "a philosophy of teaching that provides equal opportunities for all students to have a successful learning experience." However, more complex definitions exist, and those define inclusive teaching, or deep teaching, as a radical transformation of education as we know it, a movement away from teaching science to teaching students (e.g., Dewsbury, 2020; Dewsbury & Brame, 2019). Although we view the newer, more radical definitions by Dewsbury and colleagues as the ultimate goal for biology education, this manuscript does not tackle that paradigm shift. Here, we provide initial steps for how to increase active learning and inclusivity in the classroom. Aspects of inclusive teaching can be accomplished in multiple ways, including via universal design for learning, by practicing cultural competence, by using trauma‐informed practices, and by management of attitudes and expectations. It should be noted that these inclusive teaching strategies are not mutually exclusive and intersection among the frameworks does exist; however, each of these strategies has its own literature, so we presented them separately in this manuscript. In practice, these strategies overlap, and some recommendations fulfill more than one strategy.

#### 1.3.1 Inclusive teaching through universal design for learning

One approach to inclusive teaching is the implementation of **Universal Design for Learning (UDL)** which is a pedagogical approach that maximizes learning for all students. UDL suggests providing multiple access points to the material, multiple modes of expression to demonstrate learning, and an emphasis on engaging learners so that students are motivated (Dell et al., 2015). The goals of UDL are to develop expert learners who are (a) purposeful and motivated, (b) resourceful and knowledgeable, and (c) strategic and goal‐directed (http://udlguidelines.cast.org/), which are admirable goals for any classroom. Although the origin and purpose of UDL is accessibility for individuals with disabilities, this pedagogical approach can enhance the learning experience for all. Importantly, individual accommodations for students are not the purpose of UDL; rather the focus is to design a course to be accessible to all so that accommodations are unnecessary (Tobin & Behling, 2018).

Beyond the technical aspects of UDL such as closed‐captioning, fonts that are easiest for all readers, color palettes that take color‐blindness into consideration, and simple and consistent slide structures that are easy to follow, UDL emphasizes that all students learn differently. Therefore, to be inclusive and maximize learning for all, courses should capitalize on student variation by providing multiple approaches to facilitate learning. To produce learners that are “purposeful and motivated,” UDL suggests engaging all students by helping them be motivated to learn about the topic. One way of accomplishing this through active learning is to provide several primary sources rather than a traditional lecture. This way students can select the approach to a topic that is most engaging. Choice will improve motivation and has the added benefit of allowing students to avoid material they perceive as threatening and might distract from the learning goals. To produce learners that are “resourceful and knowledgeable,” UDL suggests using an array of media choices for students to access information. For instance, in the above example, in addition to providing multiple primary texts, a podcast or TED talk could be included as options to cover the same topic. Finally, to develop students who are “strategic and goal directed,” UDL suggests providing options for students to demonstrate how they learn through action and expression. To do this, variable outputs can be proposed for students to demonstrate what they learned. For example, students could choose to generate a graphical abstract for a paper they read or construct a follow‐up experiment in the form of a research proposal based on a podcast or TED talk. Allowing students to work either independently or as a group is another way to provide choice to students and enhance learning.

Regardless of the activities chosen or assignment options provided, instructors should be clear in their goals and expectations. That is, they should use transparent teaching strategies by providing the task (what students are asked to do), purpose (why they are doing it), and criteria (how they will be graded) for every assignment or activity (for more see, the Transparency in Learning and Teaching Project: www.tilthighered.com). This approach is easy to incorporate into class assignments or activities, it aligns with UDL goals, helps to remove the effects of “unwritten rules” or “hidden curriculum” often present in academia, and promotes inclusivity. All students benefit from transparent presentation of assignments, but gains are highest for first‐generation students, low‐income students, and students from groups traditionally underrepresented in science (Winkelmes et al., 2016). Paying close attention to course design, goals, and expectations is particularly important in an online course where, traditionally, students have little to no personal guidance from the instructor and need to navigate the online platform independently (Darby & Lang, 2019; Garrison et al., 1999).

#### 1.3.2 Inclusive teaching through culturally responsive pedagogy

Culturally responsive*or* **culturally compentent** **pedagogy** describes teaching approaches in which students’ different cultural experiences enrich the course material (Ladson‐Billings, 1992, 1995). Three pillars of this pedagogy include high expectations for all students, recognition and appreciation of different cultural experiences of students (cultural competence), and development of “critical perspectives that challenge inequities that schools (and other institutions) perpetuate” (social justice; Ladson‐Billings, 1995). As instructors, we aim to meet students at their level of knowledge and facilitate their learning to get them where we need them to be. To put a culturally responsive spin on this same idea, instructors should also seek an understanding of students’ lived cultural experiences to get an idea of who they are and use this knowledge to provide engaging and relevant curriculum. Classrooms that embrace culturally responsive teaching offer a conduit through which students can appreciate their own culture as well as the cultures of others (Ladson‐Billings, 2014). When diverse perspectives are recognized and valued this promotes open communication, mutual respect, and inclusion. An added focus on culturally responsive teaching within active learning is critical to create a safe space for students where they feel valued for the knowledge and lived experience they bring into the classroom.

There is no one right way to create a culturally responsive curriculum. A recent review of literature in the field of culturally responsive teaching outlines some useful principles (Morrison et al., 2008). Many of these principles aim to establish a safe, cooperative learning space for relationship‐building between instructors and students, as well as among students. Rather than the traditional “sage on the stage” model, instructors engaged in culturally responsive teaching share the stage with their students, because students’ strengths, lived experiences, and knowledge are used as starting points for instruction. Successful implementation also involves instructors who take the time to establish meaningful relationships with students and take a personal stake in their students’ success.

The task of creating an environment conducive to culturally responsive pedagogy may seem overwhelming at first, especially if one teaches high‐enrollment courses, but steps for how to build a cooperative learning environment exist. First, instructors need to honestly assess their biases (e.g., https://implicit.harvard.edu/implicit/takeatest.html) and determine how biases may affect their interactions with students. Such biases may unintentionally alienate students in the classroom in ways such as a lack of diverse curricular examples/models or presumptive language (Tanner & Allen, 2007). Critical self‐reflection on the part of the instructor is needed. Howard (2003) provides some key questions that instructors can use as a starting point for this reflection: (a) How frequently and what types of interactions did I have with individuals from racial backgrounds different from my own growing up? (b) Who were the primary persons that helped to shape my perspectives of individuals from different racial groups? How were their opinions formed? (c) Have I ever harbored prejudiced thoughts toward people from different racial backgrounds? (d) If I do harbor prejudiced thoughts, what effects do such thoughts have on students who come from those backgrounds? and (e) Do I create negative profiles of individuals who come from different racial backgrounds? Although these self‐reflection questions focus only on race, it is beneficial for instructors to think of other classifications such as gender, religion, ability status, sexual orientation, or other axes of minoritization that can be substituted here. Only after biases are acknowledged can instructors begin to take on the work of creating a more inclusive classroom.

Culturally responsive pedagogy has been a recent focus of the AAC&U through their Teaching to Increase Diversity and Equity in STEM (TIDES), which sought to aid institutions in developing teaching strategies that help STEM faculty adopt culturally sensitive pedagogy (Mack, 2019). Some principles born from this effort that were implemented in face‐to‐face courses (Hughes‐Darden et al., 2019) can provide a more descriptive framework to translate cultural competence to the realm of online teaching. These principles are as follows: (a) Incorporate physical and hands‐on activities in instructional practices; (b) Incorporate more student‐led discussions and teaching opportunities in class; (c) Become aware of personal biases and judgments that shape perceptions of students and being willing to change these perceptions; (d) Have student‐led teaching and learning apprenticeships that foster empowered learning communities; and (e) Use students’ lived experiences as a context for course content and activities. All five of these principles can apply to the online learning environment with perhaps a slight modification to principle one. These instructional practices now become student‐led and instructor‐facilitated using one or more of the active learning tools described in this article.

##### Cultural competence in ecology—an example

A recently published case study (Miriti, 2019) reviewed literature on the role of nature in ecology and environmental sciences with a focus on delineating any cultural biases. This work provides a compelling discussion on how the lack of diversity in these fields impacts discipline priorities. Miriti posits that because there is a low percentage of students and faculty from minoritized populations in ecology and environmental sciences, commonly held views on nature have been mostly dictated by those in majority groups (typically white, middle‐ or upper‐class males), which has led to exclusion of other voices. This Anglo‐Eurocentristic view has created biases in how underrepresented groups are perceived to value nature. One common misconception, for example, is that those from minority groups do not value the environment. However, the real issue lies with the Anglo‐Eurocentristic view or definition of what valuing the environment is or should look like (i.e., visiting a national park); those with diverse experiences that lie outside of this definition can be mischaracterized. In essence, this further marginalizes those who are already underrepresented in the ecology and environmental sciences and can decrease retention. Miriti suggests that by incorporating cultural competence and active learning techniques, educators and practitioners in the field can increase inclusivity allowing diverse voices to be heard.

#### 1.3.3 Inclusive teaching through trauma‐informed pedagogy

From the pandemic and social distancing to police brutality and ensuing protests, the events of 2020 have exposed even the most sheltered students to trauma. As with other social issues, the level of trauma is higher for students of color and those living in poverty. As instructors, we must acknowledge that each student's experience of trauma will differ, and that students will bring their trauma to the classroom (for more details, please see McInerney & McKlindon, 2014 and the National Child Traumatic Stress Network Learning Center https://learn.nctsn.org/). Thus, we should design our courses with student trauma in mind. The idea behind **trauma‐informed teaching** is to enable student success by interacting with students in such a way as to forge meaningful connections, allowing the students to perceive that the instructor is available and willing to assist in managing the traumatic emotions (Newhouse, 2020). For example, many individuals experiencing trauma find comfort in structure, and trauma‐informed teaching suggests the importance of maintaining a routine schedule for students exposed to trauma (Pat‐Horenczyk et al., 2006). However, other students may withdraw or find difficulty completing routine tasks. Therefore, a clear schedule with accommodations written out for late work is a way to meet both of those needs. Fostering connection and a feeling of safety is also important for trauma‐informed pedagogy. A shared narrative between students and the instructor can be useful in establishing this. For example, an instructor acknowledging their own children during a synchronous class can show the students that their instructors are struggling to cope as well and understand the burdens their students may be experiencing. Instructors can also model naming emotions by sharing their honest feelings with the class, for example by letting students know this transition is challenging for all of us and that it is okay to feel angry, sad, and frustrated. Instructors can model resilience and build community by telling students we can get through this together (Teaching Tolerance Staff, 2020). It can also be important to let students see our authentic selves on screen, not every video has to be perfect (Darby & Lang, 2019). Accomplishing this sense of community can be challenging in an online environment where students can feel isolated and alone. Thus, it is important to have some form of personalized communication, either via instructor‐created video, live online sessions, personalized emails, discussion posts, or other class activities, during the online course.

#### 1.3.4 Inclusive teaching through management of attitudes and expectations

The growth mindset framework, namely the belief that intelligence can be developed and improved and that is it not fixed (mindset theory), has been linked to multiple outcomes, but most work focused on educational gains and performance. The pedagogical literature suggests instructors should encourage a growth mindset in students (Dweck, 2015). A recent meta‐analysis examined if growth mindset was associated with increased academic performance and if growth mindset interventions improved performance (Sisk et al., 2018). The authors found weak but significant associations: Students with growth mindsets showed higher academic achievement and interventions to increase growth mindset can be successful (Sisk et al., 2018). Interventions benefited students from low socioeconomic status households and at‐risk students, but not students from middle‐ and high‐income households (Sisk et al., 2018). Mode of intervention mattered. Successful intervention involved out‐of‐class readings on growth mindset followed by writing a reflection; however, results on intervention success should be interpreted with caution as many suffered from methodological issues (Sisk et al., 2018). Another study found student commitment to active learning was influenced by growth mindset and trust in the instructor, but only the latter was related to performance in the course (Cavanagh et al., 2018). These results highlight the importance of the student–instructor interaction and are particularly relevant for taking active learning online. Instructors can increase student trust by being transparent in purpose and goals (see Section 1.3.1), showing students evidence‐based benefits of active learning, being consistent and clear in alignment between activities and assessments, and encouraging growth mindset (Cavanagh et al., 2018).

Instructors can also help students maximize the benefits of active learning by pushing students to think metacognitively about their studying and learning (McGuire & McGuire, 2015) as metacognitive thinking benefits performance. For example, students randomly assigned to complete a self‐guided online questionnaire asking them to strategize about upcoming examinations performed better than students assigned to the control (no prompt to strategize) condition (Chen et al., 2017). In addition to the focus on metacognition, instructors can help their students practice emotional regulation and enhance personal connection to the material. These practices are especially relevant for minoritized or underserved students. Students with low success expectations, low subject interest, or low self‐efficacy tend to not perform as well in academic courses and are more likely to drop out or change majors. Alarmingly, gender, ethnic, or racial performance gaps can be exacerbated in online courses (Xu & Jaggars, 2014). But, simple interventions can help increase interest and expectations. For example, Hulleman and Harackiewicz (2009) conducted a randomized trial and found that by simply asking students to write about the usefulness and utility of the science material to their own life, versus writing a summary of the science material (control group), increased self‐reported course interest and course performance (grade), and this effect was only present for students with initial low success expectations. Hood et al. (2020) found that the use of active learning in a community college anatomy and physiology course *decreased* self‐reported self‐efficacy but only among nonwhite, first‐generation students. This outcome may have been driven by anxiety, as first‐generation students rated multiple active learning techniques as more anxiety‐provoking than did continuing‐generation students (Hood et al., 2020). Luckily, a quick intervention may be able to prevent this outcome. In a recent study, Rozek and colleagues show that a simple writing intervention aimed at reappraising and dealing with pre‐examination stress improved examination scores and passing rates specifically for low‐income students in a high school biology course (Rozek et al., 2019). When moving to online teaching, implementing self‐paced interventions which ask students to consider the types of questions they think they will see, the resources they will use to prepare, and how they will use those resources (Chen et al., 2017; Zhao et al., 2014), as well as asking them how material relates to their own lives (Hulleman & Harackiewicz, 2009), and helping students manage course‐related emotions (Rozek et al., 2019) could increase academic performance. These are simple course design elements that instructors could incorporate into their online classes.

Instructor mindset, not just that of students, is important for student success. For example, a large study (>15,000 students, 150 faculty) found that students enrolled in STEM courses taught by instructors with a fixed mindset earned lower grades than those taught by instructors with a growth mindset (Canning et al., 2019). This effect was especially pronounced for Black, Latinx, and Native American students compared to white or Asian students (Canning et al., 2019), thus supporting the ideas of stereotype threat. Stereotype threat is a psychological phenomenon which occurs when negative stereotypes about a minoritized individual's group are made salient and this realization increases doubt and anxiety and decreases performance (Steele, 1997; Steele & Aronson, 1995). Experimental research has shown that inducing stereotype threat widens achievement gaps (Steele, 1997; Steele & Aronson, 1995). Instructors can communicate their fixed mindset or other implicit biases about a group in many verbal and nonverbal ways, thus providing students with unintentional micromessages about who “belongs” in STEM and who does not (Morrell & Parker, 2013). Minoritized students are disproportionately impacted by instructor mindset at the undergraduate level (Canning et al., 2019) and field‐level belief in raw or innate ability is associated with underrepresentation of women and Black academics at the faculty level across STEM fields, including evolutionary biology (Leslie et al., 2015). Thus, the way in which instructors think about student learning abilities and the way in which they communicate with students’ matters.

These behaviors and thoughts also drive the pygmalion effect (i.e., teacher expectations predict student performance) and likely have far‐reaching effects as engagement and grades in core STEM courses serve as a gateway for who can succeed and persist in STEM. For example, students from underrepresented groups (African‐American, Latinx, Native American, Native Hawaiian, or Pacific Islander), those who have low‐income status, are women, or first‐generation college students are less likely to persist in STEM (chemistry) than are their comparable peers (white, Asian, International; high‐income; men; continuing‐generation) if they earn a C‐ or below, but *more likely* to persist if they earn a C or higher (Harris et al., 2020). Thus, having an instructor that believes all students can succeed and encourages all students to maximize their potential could be critical for reducing the well‐documented achievement and persistence gaps in STEM. Actions individual instructors can take to increase inclusion include the following: (a) attending to gaps in privilege and belonging; (b) acknowledging and reducing implicit bias; and (c) actively mitigating stereotype threat (Killpack & Melón, 2016). For more details on how to achieve and actively do these steps, please see Killpack and Melón (2016). Conveying support and encouragement in an online course can be challenging as there is little to no personal interaction between student and instructor. Thus, as with trauma‐informed pedagogy, it is important to have some form of personalized communication built into the online course.

One activity that can increase engagement and foster a sense of community online is use of discussion boards. Students can create content and can comment on content created by others. The discussion board is also a great place to foster instructor‐student interaction. The following questions are not specific to ecology and evolution content and could be used in a variety of courses. These types of questions can increase sense of safety, connectedness, and can encourage metacognitive thinking and can be used in combination with many other strategies provided in this paper. Possible discussion board prompts: (a) Please list one strategy or tool you are using to help yourself transition to this new format of learning; (b) Please look over the course syllabus. Then, email me something you are hoping to learn in this course and a fact or misconception that you know about one of the topics listed on our syllabus schedule; (c) Please list one thing that you've done for your physical or mental health this week. It can be something small or something big; (d) The current situation is stressful and challenging for all of us in a variety of ways. There are lots of things we cannot control. One thing we can control is our behavior and we can spread kindness. Please list one kind thing that you have done for someone else in the last week. This can be a small or a big thing. It can be for a friend, a family member, a neighbor, a stranger, a group, or an organization; (e) Please look through the learning goals pertaining to this week's material. Then, create one original (do not copy it from somewhere else—use your critical thinking) multiple choice question that could appear on the examination for that unit. This will give you all a chance to practice metacognitive thinking about the course and will give me some possible questions for the exam; (f) Use some form of creative expression to illustrate a concept from class. Creative expression is broadly defined and can be art work (any medium; submit a pic or video of item), photography, a meme, a poem, an infographic, a short skit/performance (film it), or really anything else that is creative. Please keep these tasteful (e.g., nothing offensive, derogatory) and please create an original submission; (g) Please look back through all the material we covered this semester, remember to look over lecture notes, the readings, the class activities, and the supplemental material. Then, describe the most interesting thing you learned; (h) In your opinion, what did you learn in this course that you feel really matters for you/for life? This can be subject/material based, learning‐in‐general based, or “life‐lesson” based. The term “really matters” can be defined as narrowly or as broadly as you'd like. For your answer, please list what you learned and describe why you feel it really matters (e.g., why you chose it/how it will benefit you later).

## 2 DESIGNING BETTER ONLINE COURSES: INTEGRATING ACTIVE LEARNING

### 2.1 Online course design, structure, and format considerations

Designing better online courses is a challenge that needs to be met first by understanding the classroom in which the instructor will be operating. Some universities have guidelines about whether or not a course must be **synchronous**, in which the class and the instructor are expected to meet at designated times to do lecture or other activities, or if the course may be **asynchronous**, allowing for students to view lectures or notes and complete activities on a flexible schedule. Courses that are asynchronous may need to emphasize self‐guided activities (Table 1), such as minute papers or self‐assessment. Other courses may be what are termed **hybrid courses**, in which some part of the learning experience is online. Commonly, this takes the form of lectures being viewed before class, and class time being spent on an activity (also called a **flipped classroom**). As the pandemic continues, **HyFlex learning**, in which the instructor delivers a live class to students who may be online *or* present in the room, may play an important role for students with health vulnerabilities who cannot safely return to campus.

**TABLE 1:** Some common active learning strategies and how those strategies can be adopted for inclusive online teaching. Please note this is not a comprehensive list of active learning techniques but is meant to provide a quick‐reference framework

Active Learning Technique	Description/Definition	Level of effort to implement	Best for (small, large, either) class size	Online considerations or adaptations	Inclusive teaching considerations
Clarification Pause	Taking time to pause during a lecture, particularly after discussing complex material, to get student feedback and questions.	Easy	Either	Would work best in a synchronous format, but if recording video lectures instructors could ask students to pause video to think and jot down questions or summarize concepts. Thoughts, questions, or summaries could then be posted to a question board. Could also use PlayPosit or other software to incorporate questions and pauses directly into videos.	For students uncomfortable asking questions in front of the class, this could be coupled to anonymous polling
Minute Paper	Students respond for a minute (or a few minutes) to a prompt that encourages reflective dialogue on the topic.	Easy	Either	Could be especially beneficial for gauging student understanding if the course is asynchronous. Thoughts, questions, or summaries could then be posted to a question board or to an LMS‐based journal. Could also use PlayPosit or other software to incorporate pauses directly into videos.	Could allow students to submit responses in written, audio, or video format
Think‐Pair‐Share	Students consider a question individually then compare answers and discuss with a peer.	Easy	Either	Likely best in synchronous courses and students will need a space to collaborate. This could be Google hangouts, Google docs, Blackboard groups, Zoom breakout rooms, etc.	Discussing a topic with a peer instead of with the instructor may be more comfortable for certain students and thus allow them to get their questions answered. Peer‐to‐peer teaching in an effective way to increase learning and engagement.
Clickers	A polling device or “clicker” is used to assess student understanding of concepts. Usually done during lecture and is a type of formative assessment.	Easy	Either	Many clickers can be used with video clients. Services, such as Poll Anywhere and Top Hat can integrate with slideshows.	Allowing anonymous responses can aid in student participation and can allow students to assess their own knowledge without fear. However, clicker devices or software can be expensive and/or difficult to use. Accessibility issues with various devices can also exist.
Formative Assessments	Provide opportunities to check student understanding. Can be graded or ungraded and take multiple formats. Clickers are one example.	Easy	Either	Could take advantage of adaptive or conditional release features in LMS so that students must complete a type of formative assessment (e.g., concept check or pass an ungraded quiz) prior to moving on with material. (also see clickers above)	Incorporation of multiple assignment types and learning checks increases student performance and self‐efficacy. Offering multiple graded assignments can help with overall performance as it removes reliance on a single or a few major exams.
Current Events	Incorporation of relevant, current events into the class learning experience	Easy	Either	This can be as simple as providing relevant new stories or could be more involved (e.g., by making a problem‐based learning situation or discussion out of current events)	Adding relevance of material to daily life can increase motivation and is in‐line with UDL principles. Increasing opportunities for students to bring their lived experiences to the class is important but creating a safe environment where all student views can be heard and respected is critical.
Concept or Mind Maps	A visualization of the organization among topics, ideas, and/or evidence	Easy	Either	This could be done in synchronous or asynchronous online courses. There are multiple online tools designed for creation of concept mapping. Alternatively, students could draw by hand and photograph to upload. Note, if class is large or instructor intends to grade assignments, effort level of implementation increases.	Access to online tools and ease of use can be an issue. Allowing students to show mastery of concepts visually, with a mind map, is in‐line with the UDL framework.
Group Discussion	Breaking students into smaller subsets of the class to discuss a topic or prompt	Easy	Either	Could be done as a whole class synchronously online (rooms) or discussion board (either as a whole class or in groups by using group settings in LMS). Note, format will likely depend on class size.	Managing group work and interactions can be important. Making sure groups collaborate, work together, and value contributions of all members is important.
					
Self‐Assessment	These can take a variety of formats, but involve students assessing their own work for strengths and weaknesses	Medium	Either	There are many ways this could be accomplished, but instructors could leverage tools available in LMS (e.g., journals, discussion boards, practice quizzes, self‐assessment options in Blackboard).	Could allow students to submit responses in written, audio, or video format. Self‐assessment can aid in metacognitive thinking and can help students learn how to study. Also see literature on Ungrading for tips and ideas.
Problem‐Based Learning, Inquiry‐Based Learning, or Case Studies	These take a variety of formats, but generally ask students to apply course material to solve a (open‐ended) problem or answer a series of questions.	Medium	Either	Can be done in synchronous or asynchronous courses, but students will need a space to collaborate. This could be Google hangouts, Google Docs, Blackboard groups, Zoom breakout rooms, etc. Note, logistics for large classes can pose a challenge.	Managing group work and interactions can be important. Making sure groups collaborate, work together, and value contributions of all members is important.
Peer review	Students use a rubric to assess a peer's work	Medium	Either	Many LMS have built‐in features to facilitate peer review. Note, logistically easier in small courses.	Can take advantage of LMS rubric options to help students fairly evaluate their peers. Peer review can also help students think metacognitively about the course and their work.
Peer Instruction	Requires students to apply class concepts and explain those concepts to a peer.	Medium	Either	Many LMS have options for student–student communication or interaction.	Peer‐to‐peer teaching in an effective way to increase learning and engagement. Allowing students to design their approach to the topic can increase motivation and self‐efficacy. Students may be more comfortable asking a peer a question.
Public Service Announcement (PSA)	Students create a PSA related to course content (format types vary ‐ audio, video, infographic, billboard, etc.)	Medium	Either	Assignment can take a variety of formats and could be turned in digitally via LMS. Note, grading could be difficult in very large courses.	Can take advantage of LMS rubrics to increase transparency in expectations and grading. Also allows for easy feedback options. Allowing students the option of format (e.g., video, audio, graphical) is in‐line with the UDL framework.
Persuasive letter or Op‐Ed	Students create a scientifically sound argument about a topic related to class. Paper can be framed as an Op‐Ed or letter to an elected official.	Medium	Either	Assignment could be turned in digitally via LMS. Note, grading could be difficult in very large courses.	Can take advantage of LMS rubrics to increase transparency in expectations and grading. Also allows for easy feedback options. Instructors could make the writing assignment an iterative process and allow students to make multiple edits to their draft. This drafting process could be managed in the LMS. Could also incorporate peer review.
Experimental Design and Experimentation	Students design and/or perform experiments related to class content	Medium to Difficult	Small	Virtual labs can be challenging but there are several opportunities for conducting experiments online. Students can easily design experiments and make predictions in an online setting. They can also analyze data that were previously collected. If resources are limited, focusing on design and research methods can be beneficial.	Doing virtual labs may aid in participation as not all laboratory spaces are accessible for all students. Allowing students to show mastery of material via proposed experiments or interpretation of data is in‐line with UDL principles.
Course‐Based Undergraduate Research Experience (CURE)	Course‐based research project that engages whole classes of students in addressing a research question or problem that is of interest to the broader scientific community	Medium to Difficult	Small (≤ 25)	Works best with a synchronous format. Depending on the project, external digital imaging equipment may be necessary such as document camera, handheld microscope or digital camera.	Access to high speed Internet to allow for online synchronous learning.
Wikipedia Assignments	Term‐long series of assignments that involve the assessment, critique, editing and contribution of content (information and media) to either existing or newly created Wikipedia articles	Medium to Difficult	Small	Online and asynchronous with some synchronous meetings (optional) to support students and for students to have discussions with peers.	Possible with the right scaffolding, course dashboards already include some tools and are very customizable to meet course needs.

When creating online course content, instructors should be deliberate in their selection of materials and assignments. Using the UDL framework to create original content or to select existing content is critical. This framework is also important for creation of an online space or page as instructors should think carefully about how students will interact with the interface. Additionally, using backwards design and scientific teaching practices to develop online modules and content will aid in making the course cohesive and easy to follow. When creating online assignments, using transparent communication about what students are asked to do, why they are asked to do it, and how they will be assessed can aid in student success and in building a trusting online classroom. Not all online teaching styles allow for interaction between faculty and students. Finding meaningful ways to authentically communicate with students is important, especially for culturally competent teaching and trauma‐informed teaching. Conveying growth mindset and metacognitive learning techniques to online learners requires instructors to carefully choose their communication mode (e.g., written, audio, video) and their conveyed message. Lastly, many aspects of inclusive teaching focus on student motivation, engagement, and belonging, and this should be considered when developing online courses. For some tips on how to engage and connect with students in an online environment, see recommendations by Darby (2020).

Creating an engaging online environment can be done with any of the above delivery modes, but how this is done will likely differ. For example, asynchronous delivery of online lecture video can be effective and engaging for students, but the method of presentation impacts student engagement and satisfaction (Choe et al., 2019). Choe and colleagues tested 6 methods of online lecture video creation in an upper division physiology course and found that although scores on summative assessments did not differ following the 6 styles, student satisfaction and rating of effectiveness of styles did (Choe et al., 2019). Instructors may want to focus on video design that highlights personal connection and engagement as these factors were rated most highly by students (see Choe et al., 2019 for more details and examples); these are also important aspects of inclusive classrooms. Additionally, courses combining synchronous and asynchronous approaches align with the UDL framework, give students the opportunity to select the modality that best fits their situation (Zydney et al., 2020), and can increase engagement and motivation. Lastly, although online laboratory courses may lose the actual hands‐on component, through the application of active learning principles (Table 1), students can still apply, analyze and evaluate, and instructors may even be able to incorporate activities allowing students to fully implement Bloom's taxonomy, and create new knowledge (see Section 3.2).

### 2.2 Equity concerns in online learning

Online instruction has the potential to increase access to education (Chou & Liu, 2005) and encourage class participation by underrepresented students. However, we must acknowledge that the rapid transition to online learning in spring 2020 left many students behind. The technology itself, such as Internet and computing requirements, can be limiting for students without adequate financial resources. Even once technology is made available, at‐home‐learning requires space, familial support, and freedom from distractions (both external and internal). The pandemic highlighted the broad systemic inequalities making virtual education challenging for many. For example, a recent National Bureau of Economic Research working paper showed that low‐income students were more likely to delay graduation and to decrease time spent studying when compared to high‐income students (Aucejo et al., 2020). These issues will persist in the fall and instructors should consider limitations and equity when developing their online courses.

#### 2.2.1 Equity issues: student access to computers, Internet, and software programs

One concern for inclusive remote teaching is student access to computers and software tools. Many low‐income students lack a personal computer or may be sharing one with others trying to work from home. Many universities provide site licenses for common software tools for enrolled students. However, these may be limited to computers that access the campus wireless network—that is, a desktop computer in the student's home or place of business may not be covered. In this case, the instructor may consider use of cloud‐based tools or platforms. **Cloud‐based platforms** refer to a situation in which software is hosted on a server and not the student's computer. Google Sheets is an example of a cloud‐based equivalent to Microsoft Excel or Apple Numbers. With access to their Google account, students can access the software and perform their activities regardless of what computer they are using. Similar services exist for other data analytical applications, such as RStudio Server for courses using R or JupyterHubs for courses using R or Python for data analyses. If the university has a Microsoft 365 affiliation, this is another option for cloud‐based programs.

Instructors must also consider that, although open‐source or cloud‐based tools are free, access to a stable Internet connection is necessary to use these tools (Sarvary & Gifford, 2016). If the only way a student can access the Internet is through a mobile device, are the tools mobile‐compatible? Internet availability can be a challenge to some students who reside in rural areas or in large communal living situations with limited bandwidth. Additionally, from a socioeconomic standpoint, students may not have access to reliable Internet due to the inability of their families to afford it. The COVID‐19 pandemic is likely to amplify this disparity due to the economic downturn. Some students may have the ability to make a phone a “hot spot” for Internet. Others may need to use public spaces (e.g., coffee shops, public library) which may prove difficult if pandemic‐related social distancing protocols are still in place. Some Internet providers gave COVID‐19 breaks to students and families during spring, 2020, but it is unclear if those affordances will carry into fall. At the institutional level, there needs to be a concerted effort to assess the needs of students and make sure every student has computer and Internet access. At the course level, clear and explicit technology expectations of the course should be shared with the students prior to enrollment and should be included in the syllabus and in the course description, if possible. Lastly, it will likely be advantageous for instructors to poll students prior to the start of the course to assess individual technology access and limitations, this information can be used to make adjustments or find alternatives.

#### 2.2.2 Equity issues: mode of delivery

Flexibility in online course structure may help alleviate some equity concerns. Synchronous teaching has been demonstrated to benefit students with disabilities (Dahlstrom‐Hakki et al., 2020) and the regular schedule may help students recover from emotional trauma (Pat‐Horenczyk et al., 2006). However, synchronous learning demands that students be present on a predetermined schedule, which may be unrealistic for students caring for family, those who are sick (either with COVID‐19 or other illness), those who share a computer, or those who need to work during class times due to pandemic‐induced financial instability. Internet connectivity and bandwidth limitations can also be challenging for students in group living arrangements (apartments, dorms) or with housemates who are also working/learning online during business hours. Unfortunately, many universities are requiring faculty and students to attend synchronous classes during fall 2020, which raises serious equity issues. How will universities manage the justifiably high absentee rate for “required” synchronous classes? How will institutions address the likelihood that these absentees are more likely to be students from marginalized groups? How will institutions address issues with on‐ or off‐campus Internet access or stability?

#### 2.2.3 Equity issues: accessibility of online material

Another aspect of inclusive teaching that can pose a challenge for online learning is accessibility of materials. If instructors use the UDL framework to design their course many of the access issues will have been addressed. When including posted materials such as PowerPoint slides, pdfs, or word documents, these files should be accessible by a screen reader and need to be formatted for this purpose. For example, images that are included should have text descriptions and should use templates that are compatible for screen readers. Microsoft and Google Slides have tutorials available for designing screen‐reader friendly files and PowerPoint has an Accessibility Checker function under the Review tab. Additionally, some learning management systems can help instructors comply with accessibility. For example, Blackboard now offers an accessibility feature icon which provides a quick rating of file accessibility. If instructors would like to provide video lectures or tutorials for online viewing, that material must be accessible for all learners. For American Disabilities Act compliance, this accessibility includes closed captioning of material or providing a downloadable transcript of the presentation. There are several options for recording online content, some of which provide transcription services (voice to text) either for a fee or free of charge, others provide the option for instructors to type out text or to upload a transcript of the presentation for closed captioning. Or, universities may subscribe to an automated captioning service; this information is commonly available through the student accessibility office or teaching center. A collection of some online presentation programs are listed here: Adobe Presenter; Animoto; Blackboard Collaborate/Collaborate Ultra; Camtasia; Capture; Conceptual Academy; Final Cut; Google Slides; iMovie; Kaltura; Loom; OBS; Panopto; Powerpoint narration capture; Sapling; Screencast‐O‐Matic; Screencastify; ScreenRecorder; Snagit; Vidgrid; Voicethread; YouTube; Zoom; Zubtitle. For some practical tips and examples on how to make both laboratory and lecture content more accessible see (Hackl & Ermolina, 2019).

### 2.3 Integrating active learning online: challenges and solutions

With the mounting evidence of the effectiveness of active learning techniques, they are now used widely. This ubiquitous adoption has generated numerous success stories of active learning modalities, as well as information on potential challenges and how to overcome them. Below we list some common challenges when incorporating active learning and then provide some solutions for how to make implementation of these activities easier, more effective, and more inclusive in an online context. The content we provide is not an all‐encompassing list (see Brownell & Tanner, 2012; Petersen et al., 2020), but along with the quick‐reference active learning activities (Table 1) and web resources (Table 2) provided, should help instructors incorporate active learning into their online ecology and evolution classrooms.

**TABLE 2:** List of some databases and/or resources for ecology and evolution active learning content, and for general inclusive teaching practices

Database or website	Brief description	Focus	Website link
American Museum of Natural History, Ecology Disrupted	A 3‐part curriculum that contains videos and case studies about ecology research and connection to daily life. Also see the additional Ecology Disrupted materials created by teachers. Target audience is 7‐12th grade.	Ecology	https://www.amnh.org/learn‐teach/curriculum‐collections/ecology‐disrupted
Connecting Students to Citizen Science and Curated Collections	Teaching materials to implement a term‐long student research project that involves collecting, pressing, and identifying plant specimens. Uses iNaturalist and Symbiota. Includes materials for students and instructors.	Ecology	https://collectionseducation.org/
Conservation Bridge Case Studies	Videos, resources, and case studies about conservation.	Ecology	https://www.conservationbridge.org/
DIG into Data for the Biology Classroom	Datasets and activities intended to be used in the online ecology classroom	Ecology	https://qubeshub.org/community/groups/data_incubator/tiee
Ecological Society of America Online Teaching	Webpage by the ESA with tips and links to other resources.	Ecology	https://www.esa.org/programs/ecology‐education/online‐teaching/#gsc.tab=0
Enduring Legacies Native Cases, Evergreen State College	A collection of case studies that contains culturally relevant curriculum by focusing on issues faced by Indigenous peoples. Also includes helpful resources and assessment options.	Ecology	http://nativecases.evergreen.edu/
GIS in Ecology	A collection of case studies highlighting where GIS has played a role in ecological or marine biology studies.	Ecology	http://gisinecology.com/case_studies.htm
National Research Council Ecological Knowledge and Environmental Problem‐Solving	A National Academies of Sciences, Engineering, and Medicine free, downloadable pdf book with 13 case studies.	Ecology	https://www.nap.edu/catalog/645/ecological‐knowledge‐and‐environmental‐problem‐solving‐concepts‐and‐case‐studies
Traditional Ecological Knowledge and Conservation	A three‐day module for teaching Traditional Ecological Knowledge (TEK) in modern conservation. Website includes worksheets and interactive content.	Ecology	https://qubeshub.org/qubesresources/publications/1939/about?v=1
UK Center for Ecology and Hydrology	Collection of over 20 case studies on ecology, biodiversity, human health, and hydrology.	Ecology	https://www.ceh.ac.uk/case‐studies
Big History	Collection of free, open, online resources for teaching about the shared ancestry on Earth. Target audience is middle and high school.	Evolution	https://www.oerproject.com/Big‐History
Case It!	Molecular biology simulations for case‐based learning. A downloadable computer simulation for working with DNA or protein sequences.	Evolution	http://www.caseitproject.org/
Diversity and Inclusion in Evolutionary Sciences	BioMed Central collection of journal articles dealing with diversity and inclusion in evolutionary science.	Evolution	https://www.biomedcentral.com/collections/DIES
DNA to Darwin	A collection of 10 practical bioinformatics activities that use data analysis tools and molecular data to help students understand evolution.	Evolution	http://www.dnadarwin.org/casestudies/
Evolution Institute	The Evolution Institute is a non‐profit aimed at providing science‐based solutions. They have several project links and resources. The link here goes to an article on the site titled *It is unethical to teach evolution without confronting racism and sexism.*	Evolution	https://evolution‐institute.org/it‐is‐unethical‐to‐teach‐evolution‐without‐confronting‐racism‐and‐sexism/
Evolution and the Nature of Science Institutes	A collection of classroom lessons about evolution and natural science. Targeted to high school students.	Evolution	https://ensiweb.bio.indiana.edu/
EvoEd: Cases for Effective Evolution Education	Handful of cases dealing with evolution of traits. Has a resources tab and a games/sims tab.	Evolution	http://www.evo‐ed.org/
Exploring Life's Origins	Website with open access videos for teaching about evolution of RNA.	Evolution	http://exploringorigins.org/resources.html
Gender‐Inclusive Biology	Website with multiple teaching materials that link Next‐Generation Science Standards with gender‐inclusive teaching. Educational materials cover evolution and other topics.	Evolution	https://www.genderinclusivebiology.com/lesson‐materials
Genes to Genomes	A blog from the Genetics Society of America. The one linked here is for *Understanding our eugenic past to take steps toward scientific accountability*.	Evolution	http://genestogenomes.org/understanding‐our‐eugenic‐past‐to‐take‐steps‐towards‐scientific‐accountability/
National Museum of Natural History—Teaching Evolution through human examples	Collection of 5 downloadable case studies for teaching evolution. Also includes a section on cultural and religious sensitivity.	Evolution	https://humanorigins.si.edu/education/teaching‐evolution‐through‐human‐examples
PBS Teaching Evolution	Evolution library for teachers, contains a handful of case studies and other resources.	Evolution	https://www.pbs.org/wgbh/evolution/educators/index.html
Towards a More Human Genetics Education	Webpage with resources for teaching race and genetics in the classroom, with specific attention on misconceptions.	Evolution	https://bscs.org/our‐work/rd‐programs/towards‐a‐more‐humane‐genetics‐education/
Understanding Evolution	Educational website dedicated to teaching evolutionary biology. Page contains lesson plans, activities, and multiple other resources for a variety of age groups.	Evolution	https://evolution.berkeley.edu/evolibrary/home.php
AACU Scientific Thinking and Integrative Reasoning Skills (STIRS) Case Studies	Project to increase problem solving and critical thinking skills in undergraduate classrooms. Includes several case studies and additional resources.	Ecology and Evolution	https://www.aacu.org/stirs/casestudies
Biology Corner	A website with multiple lesson plans and resources. See the "classes" tab on the top right for course‐specific resources. There are ecology and evolution units for AP and various Intro Bio courses. Each unit has various resources including case studies and datasets.	Ecology and Evolution	https://www.biologycorner.com/
BioQUEST Curriculum Consortium	Database of investigative case‐based learning activities. Also includes many other resources. Many biology topics covered.	Ecology and Evolution	http://bioquest.org/icbl/cases.php
FlyBase	A database of *Drosophila* genes and genomes.	Ecology and Evolution	https://flybase.org/
GenBank	A publicly available database of NIH genetic sequence data.	Ecology and Evolution	https://www.ncbi.nlm.nih.gov/genbank/
Harvard University Herbaria & Libraries	The digital collection of herbarium samples. Digitization is currently underway, goal is to have a digital record of the over 5 million specimens.	Ecology and Evolution	https://huh.harvard.edu/pages/digital‐resources
HHMI Biointeractive	Several cases, laboratories, and other resources. Page also available in Spanish. Professional development links and can sign up for an account and newsletters. Has specific online education content.	Ecology and Evolution	https://www.biointeractive.org/
HormoneBase	A comprehensive freely available database of un‐manipulated measures of plasma glucocorticoids and androgens from free‐living, adult vertebrates.	Ecology and Evolution	https://hormonebase.org/
iDigBio	Integrated Digitized Biocollections (iDigBio) is part of the National Resource for Advancing Digitization of Biodiversity Collections (ADBC) funded by NSF. The collection contains electronic data and images for millions of biological specimens.	Ecology and Evolution	https://www.idigbio.org/
Knowledge Project	Archive of hundreds of articles created through "knowledge wiki". Several topics are covered, including extensive resources for ecology and evolution. Resources are categorized as basic, intermediate, or advanced level.	Ecology and Evolution	https://www.nature.com/scitable/knowledge/ecology‐102/
National Center for Case Study Teaching in Science	A database of nearly 900 case studies spanning multiple disciplines. Membership required to view answer keys and teaching notes, cases are freely available. Cases can take 1 of 15 different formats.	Ecology and Evolution	https://sciencecases.lib.buffalo.edu/
National Center for Science Education	Website run by NCSE which contains lesson plans for climate change, other resources also available. Target grade level is 5‐12.	Ecology and Evolution	https://ncse.ngo/supporting‐teachers/classroom‐resources
National Geographic Education Resource Library	Collection of thousands of educational resources and activities for teaching a wide range of subjects. Can filter by subject and grade level.	Ecology and Evolution	https://www.nationalgeographic.org/education/resource‐library/?q=&page=1&per_page=25
National Ecological Observatory Network (NEON)	A collection of long‐term, open‐access ecological data. Contains over 175 open‐access data projects. Great resource for data analysis assignments.	Ecology and Evolution	https://www.neonscience.org/
NGSS Biology	A website with multiple biology unit lesson plans, including one on Ecology and one on Evolution & Natural Selection. Many other topics and critical thinking resources. Target grades K‐12	Ecology and Evolution	https://www.ngsslifescience.com/
Network of Conservation Educators and Practitioners (NCEP)	A set of teaching modules on conservation and biodiversity	Ecology and Evolution	https://ncep.amnh.org
SERC (Science Education Resource Center at Carleton College)	Expansive website with a multitude of teaching resources. Lesson plans for Ecology and for Evolution, as well as many other topics. Contains great general information on teaching as well.	Ecology and Evolution	https://serc.carleton.edu/index.html
SHiPS Resource Center Case Collection	Cases aimed at integrating history, philosophy, and sociology in the science classroom.	Ecology and Evolution	http://www.shipseducation.net/modules/index.htm
Virtual Field Trips—Arizona State University	Contains a variety of virtual field trips offering 360 navigation and embedded lessons for the user	Ecology and Evolution	https://vft.asu.edu/
WikiEdu	As per the website mission statement, Wiki Education engages students and academics to improve Wikipedia, enrich student learning, and build a more informed public.	Ecology and Evolution	https://wikiedu.org/
ACUE Inclusive Teaching Practices Toolkit	A set of free resources, including 10 inclusive teaching practices that can be immediately put to use to benefit both faculty and their students, provided by the Association of College and University Educators.	Active and/or Inclusive Framework	https://acue.org/inclusive‐teaching‐practices‐toolkit/
AAAS assessment inventory	A question database with over 1000 items; part of Project 2061. Instructors can assess student knowledge compared to national data. Helpful for concept checks or beginning of the semester inventory of background knowledge.	Active and/or Inclusive Framework	http://assessment.aaas.org/
A Dozen Suggestions for Enhancing Student Learning	A list of 12 tips for increasing inclusivity in the classroom.	Active and/or Inclusive Framework	https://www.uww.edu/learn/aboutdiversity/approachdiversity
Active Learning while Physical Distancing	A Google doc containing incredibly helpful tips for doing active learning online and in a physically distanced classroom; open to community comment. The page was initiated by Dr. Jennifer Baumgartner, Associate Professor at Louisiana State University, and the LSU LTC and POD Network.	Active and/or Inclusive Framework	https://docs.google.com/document/u/0/d/15ZtTu2pmQRU_eC3gMccVhVwDR57PDs4uxlMB7Bs1os8/mobilebasic?pli=1
Bias‐Free Language	American Psychological Association style guide for using bias‐free language. The page includes several subsections, including gender, race, ethnicity, intersectionality, ability, age, and others.	Active and/or Inclusive Framework	https://apastyle.apa.org/style‐grammar‐guidelines/bias‐free‐language/
Bio‐MAPS	Biology Measuring Achievement and Progression in Science or Bio‐MAPS, is a suite of diagnostic assessments that aim to measure student understanding across a degree program. Includes EcoEvo‐MAPS, Phys‐MAPS, GenBio‐MAPS	Active and/or Inclusive Framework	http://cperl.lassp.cornell.edu/bio‐maps
Center for Academic Success at LSU	Website for the Center for Academic Success at Louisiana State University. This page has several handy tips, but the links about organization and the study cycle are particularly useful.	Active and/or Inclusive Framework	https://www.lsu.edu/cas/earnbettergrades/vlc/virtuallearningcenter.php
Center for Applied Special Technology (CAST)	Website devoted to removing barriers and providing access to education. Webpage has several sections, including one on UDL, and provides several resources.	Active and/or Inclusive Framework	http://www.cast.org/
Center for Teaching at Vanderbilt	Website for the Center for Teaching. There are many, many helpful subsections, including Pedagogies & Strategies, Principles & Frameworks, Reflecting & Assessing, and Challenges & Opportunities.	Active and/or Inclusive Framework	https://cft.vanderbilt.edu/teaching‐guides/pedagogies‐and‐strategies/
CIRTL Network	Center for the Integration of Research, Teaching, and Learning (CIRTL) aims to promote evidence‐based teaching practices to enhance learning. Many resources and references on this page.	Active and/or Inclusive Framework	https://www.cirtl.net/about
Community of Inquiry	An interactive site dedicated to information about the Community of Inquiry learning framework.	Active and/or Inclusive Framework	https://coi.athabascau.ca/
Critical Multicultural Pavilion	Website dedicated to building equity in education. Website has equity case studies, easy‐to‐implement awareness activities, and additional teacher resources.	Active and/or Inclusive Framework	http://www.edchange.org/multicultural/
CUREnet	An online support page for all‐things CURE, including networking, development, teaching, assessment, and more.	Active and/or Inclusive Framework	https://serc.carleton.edu/curenet/index.html
Diversity: A Nature and Scientific American Special Issue	A joint special issue collection discussion persistent, misguided assumptions. The message is clear: Inclusive science is better science.	Active and/or Inclusive Framework	https://www.nature.com/collections/fegedeebec
EmbraceRace	A page dedicated to educating children, families, and communities about race. The webpage has several resources and action guides.	Active and/or Inclusive Framework	https://www.embracerace.org/about
Epic Ebook of Web Tools & Apps	A free, crowdsource guide with 250 pages of the best EdTech with videos, tutorials, guides, and more.	Active and/or Inclusive Framework	https://librarian.rocks/epicebook
FIELD Project	Fieldwork Inspiring Expanded Leadership and Diversity Project aims to make field activity in the geosciences more accessible, culturally sensitive, and inclusive by equipping field leaders with the perspectives, skills, and solidarity to address barriers in field settings.	Active and/or Inclusive Framework	https://field.berkeley.edu/
FUSE (The Forum for Undergraduate Science Education)	Online repository for best practices and dialog on undergraduate science education. Many useful links and files.	Active and/or Inclusive Framework	http://wikifuse.pbworks.com/w/page/14383803/FrontPage
HHMI Education Resources	Links to many great resources for improving undergraduate science education.	Active and/or Inclusive Framework	https://www.hhmi.org/science‐education/programs/resources#education‐resources
Higher Ed Learning Collective	A groundswell movement grown from the need to aid educators transitioning from face‐2‐face instruction to online and remote learning during the spring 2020 semester.	Active and/or Inclusive Framework	https://higheredlearningcollective.org/
National Association of Biology Teachers	Website with a comprehensive collection of links for teaching biology.	Active and/or Inclusive Framework	https://nabt.org/Resources‐Resource‐Links
National Museum of Natural History	Links to many resources for teachers, including over 100 activities. Target audience is K‐12.	Active and/or Inclusive Framework	https://naturalhistory.si.edu/education
*Nice White Parents* discussion guide	A study guide created to accompany the podcast, *Nice White Parents*. Contains several resources discussing race, racism, and education.	Active and/or Inclusive Framework	https://www.nytimes.com/2020/08/27/learning/lesson‐plans/nice‐white‐parents‐discussion‐guide.html
Oregon State University Annotated Bibliographies	As per the website, this is a collection of articles on various topics that address gender and other diversity issues in STEM disciplines. It is updated regularly and is not an exhaustive list.	Active and/or Inclusive Framework	https://advance.oregonstate.edu/additional‐resources‐and‐articles/annotated‐bibliography
Professional and Organizational Development (POD) Network in Higher Education	Organization devoted to improving teaching and learning in higher education. Various links and sections, one linked here is for Diversity, Equity, and Inclusion.	Active and/or Inclusive Framework	https://podnetwork.org/resources/diversity‐equity‐and‐inclusion‐resources/
Problem‐Based Learning Clearinghouse, University of Delaware	A webpage to help increase implementation and assessment of problem‐based learning strategies. Includes example syllabi, evaluation forms, a few cases, and common issues.	Active and/or Inclusive Framework	https://www.itue.udel.edu/resources/pbl‐resources
Project Implicit	A non‐profit and international collaboration with the goal of educating the public about hidden biases. The website provides implicit bias tests that anyone can take.	Active and/or Inclusive Framework	https://implicit.harvard.edu/implicit/
Radiolab Presents: *G*	A 6‐part podcast series hosted by Radiolab that discusses the concept of intelligence. Discusses eugenics, IQ tests, and Einstein, among other things. The episode on Einstein (Relative Genius) would be great for discussion on ability vs. opportunity and the importance of reading for fun and creativity.	Active and/or Inclusive Framework	https://www.wnycstudios.org/podcasts/radiolab/projects/radiolab‐presents‐g
SCIPS	Strategies for Creating Inclusive Programmes of Study, a web resource devoted to promotion of inclusive teaching. Contains a section on biosciences with various links and recommendations.	Active and/or Inclusive Framework	https://scips.worc.ac.uk/subjects/biosciences/
SEA Change	AAAS partner working to make diversity, equity, and inclusion in STEMM the norm. Website has various resources.	Active and/or Inclusive Framework	https://seachange.aaas.org/port‐of‐call/resources
*Seeing White* Study Guide	A study guide created to accompany the podcast, *Seeing White*, produced by Scene on Radio. Contains several resources discussing race and racism.	Active and/or Inclusive Framework	http://www.sceneonradio.org/seeing‐white/seeing‐white‐study‐guide/
Teaching in Higher Ed	Website with many resources, including a podcast series, that discuss inclusive teaching and active learning.	Active and/or Inclusive Framework	https://teachinginhighered.com/
Teaching Online Pedagogical Repository	A free database/webpage run by the University of Central Florida. The website has many entries of ready‐to‐use examples of online or blended classroom pedagogical practices.	Active and/or Inclusive Framework	https://topr.online.ucf.edu/
Teaching Tolerance	A group dedicated to help teachers and schools educate children and youth to be active participants in a diverse democracy. Includes several classroom resources, activities, and lessons as well as professional development information.	Active and/or Inclusive Framework	https://www.tolerance.org/
The National Child Traumatic Stress Network	Website devoted to raise the standards of care for and provide services to traumatized students. There is an education branch of the website with many resources and articles.	Active and/or Inclusive Framework	https://www.nctsn.org/
TILT Higher Ed	Transparency in Learning and Teaching (TILT) website aims to improve higher education teaching and learning for students and faculty. Contains several examples and resources.	Active and/or Inclusive Framework	https://tilthighered.com/

*Challenge 1: Depth* versus*Breadth*. Active Learning often necessitates cutting course content in order to make space for active learning techniques (Roach, 2014). Even though there is strong evidence that exposing students to more topics on a shallower level does not increase learning; cutting material remains difficult (Petersen et al., 2020). Reasons for this difficulty include textbook design, discipline‐specific norms, standardized exams, accreditation of programs, departmental norms, instructor priorities, and a host of other factors (Petersen et al., 2020).

*Solution:* Instead of just covering a list of topics and specific facts, Petersen and colleagues (Petersen et al., 2020) recommend focusing on core concepts and competencies and building the course content around mastery of those concepts; this practice aligns *Vision and Change* (AAAS, 2011, 2015, 2018). Excellent tools exist to help biology faculty implement this framework in their courses, for example the BioCore Guide (Brownell et al., 2014; Cary & Branchaw, 2017) and the BioSkills Guide (Clemmons et al., 2020). Both Guides provide excellent recommendations to assist in course design focused on critical concepts and competencies. Reframing course structure from a set number of chapters to a more holistic understanding of biology and critical skills provides multiple opportunities to incorporate active and inclusive practices.

In addition to making changes within individual courses, implementation of curriculum‐wide changes at the department level are also possible, discussed in depth by Branchaw et al. (2020). However, of particular relevance to this discussion are two specific Measuring Achievement and Progression in Science (Bio‐MAPS) tools. First, the general biology (GenBio‐MAPS) tool can be used at the introductory level (Couch et al., 2019). Second, the specifically targeted toward Ecology and Evolution EcoEvo‐MAPS tool can be used at an upper division level (Summers et al., 2018).

*Challenge 2: Where to get activities?* Finding or creating active learning content can be overwhelming and/or intimidating for instructors.

Solution: We recommend that instructors examine the numerous resources that already exist before creating activities from scratch. We have compiled some of those resources in Table 2. For example, the National Center for Case Study Teaching in Science (https://sciencecases.lib.buffalo.edu/) contains over 850 active learning assignments ready for free download. With a small membership fee, instructors also get access to answer keys and teaching notes for how to integrate that material into the classroom. Instructors are also encouraged to see previously published lists: ten tools for group case studies (Prud’homme‐Généreux, 2016a) and twenty‐one teaching strategies that can be implemented to increase equity and inclusion in the biology classroom (Tanner, 2013). If instructors want to create their own active learning content, it is okay to start small and to use published literature as a guide (Bean, 2011; Burrow, 2018; Handelsman et al., 2007; Herreid et al., 2016). One quick inclusivity technique that instructors can implement when choosing or creating their own content is to include diverse names and voices for characters and story lines used in assignments, and to avoid the use of specific cultural references or analogies as these can alienate students.

*Challenge 3: Overwhelming Students*. The bounty of active learning techniques and wide array of technological options creates the potential to overwhelm students and compromise the learning experience. Additionally, online tools require acceptance, familiarity, and instructor capability to guide students in the use of these tools (Sarvary & Gifford, 2016).

Solution: By intentionally choosing active learning activities and tools that support the learning outcomes of the course, instructors can promote deep engagement and maximize effectiveness (Olimpo & Esparza, 2020; Prince, 2004). This approach uses the technology to enhance teaching instead of teaching to the technology, and is further facilitated by conducting a cost‐benefit analysis. For example, if a tool will only be used a few times during the course, is it worth investing the time, energy, and money to apply it?

Previous work on effective online instruction indicates the importance of course structure, clarity of course expectations, and flexibility (Crews & Butterfield, 2014). Make the most of the LMS (Learning Management Software) tools that are already available and develop course pages that organize content by week and contain checklists and links for assignments, quizzes, and other tasks. If multiple media are used (e.g., a publisher's software site, a remote response system [e.g., Top Hat] and a collaboration platform [e.g., Google Docs)], integrate those into the course site and weekly pages as much as possible even if that simply means using an icon to depict the platform where work will take place. Additionally, as discussed above, investing class time for technology training and/or low‐stakes practice sessions to create familiarity pays off significantly.

*Challenge 4: Whose voice is heard*. The use of active learning modalities does not guarantee equal participation by all students. Status in various groups can impact student willingness to speak up in class (see Table 1 in Kim & Sax, 2009), and active learning practices that ask students to volunteer answers may promote bias. For example, in discussions following group activities, men volunteer answers and are asked to respond to questions more often than women, and men report higher science self‐efficacy than women (Aguillon et al., 2020). Moreover, students with nonbinary gender identities may fear misgendering by instructors and peers and consequently limit their participation.

Solution: Instructors should clearly articulate norms and expectations for group discussions, or even ask students to co‐create these expectations as a class. Instructors for synchronous classes can use “random call” techniques where students know that they will be responsible for answering questions or presenting their group's work. Consistent use of random call decreases student anxiety in class discussions (Dallimore et al., 2013), increases participation by women students (Eddy et al., 2014), and may improve the quality of overall student participation (Knight et al., 2016). Implementation of tools that allow students to ask questions without knowledge of their peers (e.g., chat boxes, www.incognea.to) or to present responses anonymously (e.g., Poll Everywhere, TopHat, Kahoot, Socrative) can help increase diversity of participation and reduce student anxiety. To help increase use of correct gender pronouns, when using online systems that are not anonymous (e.g., Zoom), instructors can set the norm for adding pronouns after their display name. Instructors can also use gender‐neutral pronouns throughout the course (e.g., they instead of he or she; humans or people instead of mankind; folks or folx instead of “guys”) as this practice can help reduce bias (Tavits & Pérez, 2019). Finally, instructors should acknowledge their own (implicit) biases when calling on and responding to students, as these interactions are ways in which instructors can communicate (unintentional) messages about who belongs in STEM.

*Challenge 5: Unbalanced preparation*. Students enter our classrooms with diverse levels of preparation and with various misconceptions about the materials. These differences can pose challenges for group or collaborative projects if there is insufficient course support or infrastructure (Matsushita, 2018). When students enter a task without background knowledge, they focus on “externalization,” that is simply completing the task. However, solving problems, talking with others, and writing are not evidence of deep learning unless they are coupled with “internalization” or absorption of knowledge independently and outside of class. The two must go together (Matsushita., 2018).

Solution: Using a flipped classroom model potentially addresses these concerns and is well‐suited to (synchronous) online class meetings focused on problem‐solving and interaction (Nahar & Chowdhury, 2019). In the flipped classroom setting, students perform tasks independently outside of class and then engage in active learning and collaborative activities during class. Independent preparatory activities can include worksheets, instructor lectures via video, other supplemental videos, or readings designed to help students come to class able to expand their knowledge in deeper ways by engaging with peers (DeLozier & Rhodes, 2017). In‐class assignments and activities should address common misconceptions students may bring to the class from their previous experiences or independent preparation (Prud’homme‐Généreux, 2017). When coupled with instructor feedback and/or discussion of correct answers during the session, these activities can deepen learning and correct misconceptions (Kalinowski et al., 2010; Michaels, 2006). Flipped classroom approaches vary greatly; however, across numerous disciplines, the flipped approach has significant benefits (van Alten et al., 2019). Prud'homme‐Genereux and colleagues provide helpful tips for instructors on how to choose or create videos for flipped classrooms (Prud'homme‐Généreux et al., 2017) and how to have students produce video content (Prud’homme‐Généreux, 2016b). Having students create short, video content for online learning can increase STEM self‐efficacy (Campbell et al., 2020), and thus, making student content part of the course can be beneficial.

*Challenge 6: Group work*. Active learning techniques often require group work or student collaborations, yet students tend to dislike working in groups (Taylor, 2011). In addition to student dissatisfaction, potential problems arise when not all students participate, or when groups do not know how to handle disagreement (Smith et al., 2009).

Solution: Communication and collaboration are critical competencies identified by *Vision and Change* (Clemmons et al., 2020). Additionally, diverse groups tend to produce more creative and better answers (see Dutcher & Rodet, 2018; Freeman & Huang, 2015; Jang, 2017). Transparent teaching strategies (see Section 1.3.1) that present data on the effectiveness of group collaboration can increase student buy‐in to group work (and active learning in general). Group work facilitates learning (Michael, 2006; Taylor, 2011) and enables students to practice the collaborative, problem‐solving skills needed in potential future careers. For example, many biology students are premed or prehealth and teamwork and collaborative skills are critical for these fields. Showing students data or articles related to collaboration in medicine (e.g., Lerner et al., 2009; Ranjan et al., 2015) will help them understand the importance of group work.

Giving students strategies for group management and leadership, and tools for how to professionally discuss material, can aid in group performance and make group work more enjoyable for participants. Students should also have a clear understanding of their role and responsibilities in the group. Group Work and Roles Guide and/or a Group Contract can facilitate this process (Woodley et al., 2017; link to document: http://bit.ly/CGWO2017). To help prevent groups just rushing to get the points, the focus should be on application of material and the process of learning; however, the use of points for correctness of answers can be beneficial for high‐risk students (Freeman et al., 2007). Regardless of how points associated with active learning are used, instructors should also provide students with detailed information on grading criteria and assessments of group performance (Livingstone & Lynch, 2002; Moog et al., 2006; Smith et al., 2009).

Groups can be student chosen, instructor chosen, or randomly selected, and can be short‐ or long‐term arrangements. Several studies suggest that group formation practices impact the learning experience (Adams et al., 2002; Chapman et al., 2006; Freeman et al., 2017; Lacey et al., 2020; Matta et al., 2010; Micari et al., 2016; Smith et al., 2009). If instructors are concerned about group composition, it can be helpful to assign groups. Ensuring that less‐prepared and more‐prepared students are working together can be helpful.One can form these groups by asking all students to initially self‐sort in order of past experience with the topic and then counting off; all students benefit and the less‐prepared students benefit the most (Micari et al., 2016). However, it is important not to shame students for lack of prior knowledge; if the in‐person sorting cannot be arranged in such a way that the question on which they are sorted is nonthreatening, it may be best to use a confidential survey and use that information to create groups.

Many applications and digital tools are available to help coordinate online group work. Options such as Google Hangouts, Blackboard Groups, Google Docs, Slack, and Zoom breakout rooms provide options for online group interaction. Before using a method, though, instructors should check with their university on approved electronic and information resources for their campus (for example, at one author institution certain tools, e.g., Piazza, are not permitted).

*Challenge 7: Student buy‐in*. At first, students may be hesitant or critical of the new learning techniques for a variety of reasons (reviewed in Shekhar et al., 2020). Because the active classroom shifts responsibility from the professor to the students (hooks, 1994; Silverthorn, 2006), students have to do more work. Students cannot passively sit and take notes or just listen; they must engage and interact with the material. This may be scary for students since this shift can push them out of their comfort zone and make them feel vulnerable in the classroom (hooks, 1994).

Solution: Instructors can help students feel more confident and comfortable by making the classroom experience inclusive (see Section 1.3) and by being clear and explicit in their rationale and expectations for in‐class activities (Silverthorn, 2006; Tharayil et al., 2018). They can also increase student buy‐in and reduce student resistance by using various evidence‐based techniques (Cavanagh et al., 2016; Finelli & Borrego, 2020). Here, we provide an activity (Appendix S1) to introduce students to group work and increase buy‐in to active learning. This activity was used in class by one of the authors. We encourage instructors to use and modify this activity as needed for use in their classroom.

*Challenge 8: Impact on course evaluations*. In addition to push‐back in the classroom, instructors may see more negative feedback on their course evaluations. When students were randomly assigned to an active or passive learning environment, those in the active environment learned more (determined by assessment) but reported lower perception of learning and had less overall rating of the course (Deslauriers et al., 2019).

Solution: Student evaluations of teaching (SET) should be interpreted with caution because they reflect student perception of learning and not necessarily actual learning (Deslauriers et al., 2019). These findings are important for any faculty member whose reappointment, merit, or promotion is based on SETs, but are particularly relevant for women faculty and faculty from minoritized groups. SETs are already biased against both groups, with students rating women (MacNell et al., 2015; Mitchell & Martin, 2018) and minoritized faculty (Chávez & Mitchell, 2020; Wallace et al., 2019) lower than (white) men. Instructors who use active learning, especially women faculty or faculty of color, should be proactive and explicitly mention these points in their yearly performance reviews. Department heads responsible for preparing annual evaluations of effectiveness should also take this information, and information about other aspects of racial and gender bias relevant to impacts of COVID‐19 (Malisch et al., 2020), into account.

## 3 TAKING THE PLUNGE

### 3.1 It is okay to start small

It is hard to change the way we teach (Silverthorn et al., 2006). Instructors often want to alter their teaching practices, but face barriers to making changes (Brownell & Tanner, 2012; McMurtrie, 2019; Petersen et al., 2020). Barriers range from fear of student push‐back, lack of administrative buy‐in or support, lack of incentives, impacts on merit and promotion materials (e.g., student evaluations of teaching), lack of time, lack of resources, lack of training, too many resources and feeling overwhelmed, integrating and grading active learning assignments, depth versus breadth, and others (Section 2.3).

These concerns are valid but going into fall 2020 and beyond we have an opportunity. The COVID‐19 pandemic has upended all aspects of higher education including the heart of universities—the classrooms. This situation provides us with the unique opportunity to reinvent the way we teach. We are already grappling with the switch to more online instruction—why not use this opportunity to alter the way in which we present material and engage with students as well?

We can make small changes and do not have to incorporate everything at once (Auster & Wylie, 2006; Darby & Lang, 2019; Lang, 2016; Tanner, 2013). For those ready to make bigger changes, below we provide some examples of techniques for use in ecology and evolutionary biology courses. These techniques in Section 3.2, combined with discussion prompts above, items in Table 1, Table 2, and/or Appendix S1 should give instructors a range of options.

### 3.2 Three explicit examples of online, active, and inclusive learning for Ecology and Evolutionary Biology Courses

#### 3.2.1 Wikipedia assignments

Many instructors tell students some variation of the following, “Do not use Wikipedia as a credible source, it can be edited by anyone on the internet!” The open collaboration framework of Wikipedia means that no official entity vets or monitors contributions and adherence to community guidelines coupled with reliance on multiple contributors, creates wariness as to its validity. However, it is exactly this open nature of Wikipedia that allows instructors to harness the power of Wikipedia to provide rich educational experiences. In 2013, the Wiki Education Foundation (Wiki Ed) was created with the mission to “engage students and academics to improve Wikipedia, enrich student learning and build a more informed public” (https://wikiedu.org/mission‐and‐vision/). This organization provides free resources and serves as a bridge for instructors wanting to incorporate writing or editing of Wikipedia content into their classrooms.

A major draw to Wikipedia assignments is the ease with which they can target multiple elements of Bloom's Taxonomy (Anderson et al., 2001) and the specific core competencies detailed in the BioCore (Brownell et al., 2014) and BioSkills Guide (Clemmons et al., 2020). Assignments that involve the assessment, editing, and contribution of content to the Wikipedia platform also tackle a host of the core concepts and competencies detailed in *Vision and Change* (AAAS, 2011, 2015, 2018). Wikipedia assignments span multiple levels of complexity to fit instructor needs and comfort with trying this new modality along with meeting course objectives. For example, students can be asked to create new Wikipedia articles from scratch, to edit existing articles, or to translate existing content into another language. As per guidance from the Wiki Ed team, these assignments can span a few weeks to a full term (https://wikiedu.org/teach‐with‐wikipedia/). Additionally, these assignments are highly compatible with courses in Ecology and Evolution because of the broad availability of article types that instructors and students can choose to edit.

For Wikipedia assignments, all work is completed through a Wiki Education Dashboard. The Wiki Education branch of Wikipedia builds course dashboards, offers assignment design guidance, provides staff support for students, and offers online trainings, all free of charge. The staff also supports instructors and students throughout the duration of the course. In‐line with scientific teaching and backwards design, instructors can target Wikipedia content based on course learning objectives and the BioCore guide (i.e., figure 3 in Brownell et al., 2014) and the BioSkill guide (Clemmons et al., 2020). The Wiki Ed staff work with instructors to facilitate the creation and structure of a course dashboard and assignments thus facilitating implementation and execution of Wikipedia assignments for both faculty and students (https://wikiedu.org/teach‐with‐wikipedia/). Additionally, the structure of the assignments developed by Wiki Ed is directly compatible with the BioSkills Guide and Bloom's taxonomy. A sample dashboard from H. Schutz's spring 2019 course can be found here: https://dashboard.wikiedu.org/courses/Pacific_Lutheran_University/Comparative_Anatomy_(Spring_2019). Additionally, all course and campaign dashboards can be viewed at Wiki Ed (https://dashboard.wikiedu.org/explore). All available dashboards provide useful models, but it may be particularly helpful to search for ecology or evolution course dashboards for instructors to see examples of course design and for students to see working examples of their assignment outcomes (Kilpatrick et al., 2020). To create a dashboard, instructors should contact Wiki Education staff, using the form on the webpage, at least one month prior to the start of their course.

Students engaged in Wikipedia assignments via the Wiki Education Foundation program begin with comprehensive training and small tasks. Students draft all work in their account sandboxes, pages that are publicly accessible but designated as drafting spaces. Their first assignments involve evaluation as they review existing articles for readability, grammar, and accuracy, and assess the quality of sources based on their training via the dashboard and instructor guidance. Learning to discriminate between sources based on their reliability and quality and identifying and correcting plagiarism via a hands‐on evaluative and iterative process is part of the Wikipedia training and assignments. Before ever beginning to edit content, students engage with anyone editing or monitoring the article to discuss proposed changes. Students review, comment on, and suggest changes to existing Wikipedia article entries in the article talk pages (a component of every Wikipedia article where editors discuss changes and updates to a page). In the sandboxes, students then begin to draft content and continue in evaluation mode throughout the semester, they peer review the work of other students and receive feedback on their own drafts. Students formulate revisions and plans based on peer, instructor, and Wiki Education staff feedback (again all happening in their sandboxes before going live). After engaging with students and other Wikipedia editors at large (anyone, anywhere can edit Wikipedia at any time and can engage with students) in the talk pages, students often find support for their proposed changes and execute them on the actual article page. As they begin to create content, students must break down complex ideas, often from the primary literature, and then integrate, organize, and convey this content to a broad audience. All of these elements are deeply compatible with the Communication and Collaboration elements in BioSkills. Additionally, context‐based education and training of students about plagiarism improves their capacity to identify and prevent it (Holt, 2012). Because students actively search for and correct plagiarism (as is done in the Wikipedia assignment described above), their efficacy and competence in avoiding plagiarism is much greater than if they simply read about it or participated in an online training about plagiarism (Holt et al., 2014).

Writing is one of the primary media used in Wikipedia assignments and is consistently associated with increased student engagement (Camfield & Land, 2017). In particular, the use of iterative assignments that begin early, start simply, build on one another and focus on the synthesis and summary of ideas and include peer review produces deep learning of content via communication of diverse and complex ideas (Balgopal et al., 2018). Building on low‐stakes activities stimulates student engagement and builds self‐efficacy (Camfield et al., 2020; Sawyer et al., 2017). Additionally, the knowledge that their work potentially reaches large and diverse audiences drives student's engagement and motivation (Konieczny, 2016).

Working with students on Wikipedia not only allows instructors to target concepts and competencies, but also potentially increases inclusivity in a course because of the compatibility of many of the inclusive teaching strategies discussed above with Wikipedia assignments and the dashboards accompanying a course. The assignments are hands‐on and active, as students are authors to all changes. Instructors can guide students to articles for editing or development, but often students propose their own articles to edit and they do so as part of small teams, making the effort primarily student‐led and collaborative. Allowing students to choose their own entry to the content and assignment aligns with UDL principles. The design of dashboards provides significant structure for students and their customizable nature makes incorporation of TILT and UDL principles easier. The Wikipedia guides for best practices also invite instructors to assign students a reflective piece at the end of their contributions asking them to reflect on what they learned about how Wikipedia works, how peer review worked for them, how they reacted to feedback, and how they were treated by the broader Wikipedia community. Most importantly, it asks students to reflect on the impact of their contributions. This reflection on impact encourages students to use the elements of course dashboards tracking the number of edits made, number of references added, number of media uploads and the number of article views for the duration of the course. Students often pleasantly shocked at the amount of traffic their articles receive and therefore the significant readership they have the capacity to reach often comment that these make the Wikipedia assignment more valuable to them than a term paper.

Working with students on Wikipedia does have some pitfalls that should be considered and addressed. Wikipedia is a community‐built Encyclopedia and thus anyone can contribute via adding, editing, or deleting content at any time. Most editors, the people doing the bulk of contributing, editing, and gatekeeping of posted information, are white cis‐gendered men and content often has significant ingroup bias (Oeberst et al., 2019). Content also contains both race and gender gaps (see Xing & Vetter, 2020 for a full review) as well as gender bias (see Wagner et al., 2015 for a review). These issues not only affect content quality, but also generate very real concerns about online safety and comfort that resulted in some Wikipedia editors who identify as women to leave the space (Menking et al., 2019). In the context of a course, instructors can mitigate some of these effects by suggesting that students use gender neutral account names not tied to their identities. Additionally, articles linked to a Wikipedia course dashboard are flagged as such and Wikipedia editors are encouraged to behave generously with new student editors.

In the last decade, the Wiki Education Program's partnerships with instructors across disciplines to facilitate teaching with Wikipedia resulted in a significant surge of student editors subsequently diversifying the editorial population by their very presence. Of the general Wikipedia editor population only 20% identify as women, whereas 68% of student editors do (Wiki Education, 2020; https://wikiedu.org/changing/wikipedia/). Students also diversify content either as part of their assignments or participation in various editing projects or a combination of both (Montez, 2017; Xing & Vetter, 2020). Students engage in the Science and Society competencies of BioSkills as Wikipedia assignments can include elements from the Women in Red project which aims to address the gender gap in Wikipedia entries or the recent #HackforBlackLives event aimed to improve Wikipedia content on Black academics and issues of social justice. Additionally, much like with group work, diverse Wikipedia teams often produce higher quality content than more homogenous ones (Lerner & Lomi, 2018; Sydow et al., 2017).

#### 3.2.2 Course‐based Undergraduate Research Experiences (CUREs)

In 2008, the AAC&U identified 10 high‐impact practices that increase undergraduate student performance for all, but especially for students from underserved groups (Kuh, 2008). Undergraduate research is one of these practices and the goal of the 2008 report was to make excellence inclusive, that is to empower educational goals for all students, not just for some (Kuh, 2008). Course‐based undergraduate research experiences (CUREs) provide an excellent way to make this high‐impact practice available to whole classrooms of students, instead of to just a few. CUREs reduce barriers to student research (e.g., lack of positions, limited access) and help alleviate impact of the “hidden curriculum” (e.g., networking, applying to a lab, having a CV) often present in academia (Bangera & Brownell, 2014). Thus, CUREs provide research opportunities for students from various backgrounds and can make the opportunity to do research more equitable. CUREs also address numerous core concepts and competencies detailed in *Vision and Change* (AAAS, 2011, 2015, 2018) and in the BioSkills Guide (Clemmons et al., 2020). Lastly, CUREs can be done in both introductory and upper‐level courses by adjusting learning outcomes and scope of the project (Bangera & Brownell, 2014; Shortlidge et al., 2016, 2017), making them a good option for multiple different courses, including those in nonmajors biology (Ballen, et al., 2017).

CUREs are designed to engage students through active learning by applying material learned in lecture to research questions that could impact the broader scientific community (Auchincloss et al., 2014; Shortlidge et al., 2016). According to a collective report, CUREs should (a) engage students in multiple scientific practices (e.g., asking questions, building models, proposing hypotheses, collecting, analyzing, and interpreting data); (b) contain elements of discovery, that is students should address novel scientific questions and outcomes should not be predetermined; (c) make students part of the broad scientific community, either via authorship, dissemination of findings to relevant stakeholders, or other activities; (d) involve collaboration and cooperation among students; and (e) embrace the iterative nature of science (Auchincloss et al., 2014). More details on the above points and on what makes a CURE different from a traditional lab, an inquiry‐based laboratory, or an internship can be found in the literature (see Auchincloss et al., 2014). CUREnet (https://serc.carleton.edu/curenet/) was established in 2012 to support networking among faculty developing, teaching, and assessing CUREs, and provides a wealth of information for instructors wanting to incorporate CUREs into their courses. Instructors should also see work by Shortlidge and colleagues (Shortlidge et al. (2016), Shortlidge et al. (2017)) for helpful information on challenges and solutions to creating and implementing a CURE. CUREs are typically based on an instructor's own research program and thus the type and scope of questions changes from semester to semester, so there is no single way to develop a CURE. However, CUREnet has a database of CUREs available for searching, it may be helpful to view other ecology and evolution course CUREs for inspiration (https://serc.carleton.edu/curenet/collection.html).

Due to the hands‐on nature of scientific research, it may initially seem difficult to successfully implement a biology‐based CURE online; however, with a strong focus on key learning outcomes and careful course design, it can be done. By using scientific teaching and backwards design, instructors can distill the critical aspects of their CURE framework and make sure online activities emphasize those points (Cooper et al., 2017). They can then define the research questions and the scope of work appropriate for their course name and student level. For a helpful flowchart on how to organize and structure a CURE, see work by Cooper and colleagues (especially see Figure 1 in Cooper et al., 2017).

One of the major learning outcomes for CUREs is to have students participate in iterative work, and in the process gain problem‐solving and critical thinking skills (Cooper et al., 2017). CUREs also aim to increase scientific literacy, encourage pro‐science attitudes, and build evidence‐based, decision‐making skills (Cooper et al., 2017). The online format does not allow for students to participate in direct laboratory experimentation; however, they can still engage in many aspects of a research project that do not require use of equipment or access to research sites. Most CUREs take place during a course laboratory session, which is usually a period separate from the lecture. With the transition online, the entire time slot allotted for the laboratory may not be feasible. Therefore, one of the first adjustments to move CUREs to an online format is to tailor the scope of research questions addressed as the course's main goal to fit in the possibly shortened class session time frame of the online format.

The research questions presented to students should be scaled down to be achievable in the time frame of a semester and with the switch from bench or field work to more computational or data analysis techniques (see Section 3.2.3). In a traditional CURE, the research questions presented to students are individualized and involve numerous laboratory techniques, the questions in an online format must be adjusted due to inability to physically access the laboratory. The research question(s) should be small in scope and engage students, but also be achievable in the realm of a semester (Auchincloss et al., 2014). It is not necessary for students to tackle a large research project, and it is incredibly difficult due to the semester time limit (Cooper et al., 2017). It is appropriate in CUREs for students to work in small groups and address specific aspects of the same research question. The small project size does not affect student's ability to achieve the course learning objectives. Use of an online video conferencing tool and synchronous meetings are necessary to aid the collaborative nature of research. As to not overwhelm students, each class or CURE laboratory session should only focus on only a few of the course's learning objectives at a time while simultaneously meeting the broader CURE learning objectives mentioned above. Therefore, meetings can be categorized into three types: (a) student‐led primary literature readings (also known as “journal clubs”) to increase scientific literacy, (b) videos or live demonstrations of experimental techniques/field work and (c) analysis of primary data collected by the instructor or other researchers in the field to build evidence‐based decision‐making skills. All three of these class session types provide an opportunity to encourage pro‐science attitudes.

The first type of class session, primary literature readings, requires students to choose research articles that provide background information for their individual research question. Project options should be provided at the start of the semester to give students ample time to decide on their direction. Faculty members should provide students with a small list of research question options and act as guides as they progress. This way students are entering the course interested in the project, but able to have a sense of supervision. Students present important findings from research papers that directly relate to their research question. This will provide background and significance for their specific project. Allowing students agency over articles and research avenues is in‐line with UDL and can help increase motivation. It is recommended that the instructor organize the first class session to provide an example of organization and scope of each presentation.

The second type of meeting, live demonstrations of experimental techniques/field work, requires the use of external devices such as document camera, video camera, or handheld microscope. The use of technology in a CURE is even greater than a traditional laboratory as CUREs emphasize the experience, use, and application of laboratory techniques (Auchincloss et al., 2014; Shortlidge et al., 2016). Use of the technology is even more accentuated in an online format. Incorporating external technology provides the opportunity to bring students closer to the experimental details. It is recommended if you are videoing a complicated fieldwork protocol to employ a second person for filming. The advantage of creating content live or during a synchronous session is it can be recorded and most, but not all, video conferencing programs will provide a transcript of recorded sessions keeping the course accessible and inclusive. This will aid any students who cannot attend the live session or those who have hearing difficulties. Also, it allows students to ask the instructor questions during the demonstration and provides instructors the opportunity to engage in discussion with students and adjust technique explanations if needed, thereby keeping the session more collaborative. The instructor can incorporate techniques incrementally through the semester starting simple and building on the techniques making it an iterative process. If it is not possible to create content de novo, using other researchers' videos is perfectly acceptable and continues to fulfill the objectives of the course. If utilizing videos made by other researchers try to choose videos that are concise, shorter in length, only cover the techniques directly related to the project and provide captions or a transcript (for helpful tips for using videos in classes, see Prud’homme‐Généreux et al., 2019).

The third type of class session, data analysis, allows students to gain critical thinking skills and apply their knowledge from previous meetings. Students can gain practical knowledge of how data is compiled, interpreted, and evaluated by professional researchers. Giving students the time to explain results and create figures contributes to their overall understanding of research and improves their skills as scientists. Continued formative assessment throughout the CURE is recommended to gauge student comprehension of specific learning objectives. With application of the aforementioned techniques, online CUREs are a powerful tool to expose students to research and to address core competencies in evolution and ecology. Once students have analyzed data and drawn conclusions about their dataset and research question, they can prepare that material for broader dissemination. Instructors may encourage students to prepare manuscripts for submission to traditional peer‐reviewed journals, or to places like Science Matters Journal, BMC Research Notes, Micropublication.org, or the Journal for Young Investigators. However, dissemination can be accomplished in a variety of different ways and will depend on the course, scope, and project.

Non‐laboratory‐based student research can also be used to increase inclusion in the current classroom and the future curriculum. For example, Favero and Van Hoomissen created a new course in which anatomy & physiology students were tasked with the creation of culturally relevant examples in human biology (Favero & Van Hoomissen, 2019). To do this, students had to locate, organize, read, and synthesize literature. They also had to apply that knowledge to create new content. The ultimate goal was to create diverse and inclusive teaching material for future courses, but the experience provided much more than the original goal, for students and for instructors. This application of student‐led research engaged and motivated students and met several of the core competencies listed in *Vision and Change* and in the BioSkills Guide. Instructors can use assignments like these to continually interrogate racist, sexist, heteronormative, Eurocentric information presented in many textbooks and to diversify their curriculum and syllabus. This type of course‐based research could easily be incorporated into an online ecology or evolution course and diversification of the curriculum in these fields is urgently needed.

#### 3.2.3 Data analytic activities

Another active learning concept that can be implemented remotely is data analysis. Data analysis targets the Analysis level of Bloom's taxonomy most naturally, but also Evaluation (synthesis of new knowledge from data) and Creation (suggestion of further research). Data analysis can range from performing calculations in a spreadsheet program, such as Excel, to performing complex modeling in advanced software packages.

As with other active learning components discussed above, the most important consideration for data analytics is to decide how best to support the course learning objectives. Much as in CUREs, another consideration is how to support skills development. This will inform what precise software platforms will be used for student activities. For example, in many ecology and evolution studies, use of Excel is perfectly adequate. However, in DNA sequence analysis, Excel is not a useful platform. Wright et al. (2019) lay out a number of considerations for choosing software, including consistency with what other instructors are using, instructor comfort with the tool, and the current status of research tools in the field. For example, if analyses in the discipline are commonly performed in R, and other courses in the curriculum are taught using Excel, the instructor might go with either methods depending on their comfort with the two tools. See Section 2.2.1 above for more on access issues to data analysis tools and software.

One further consideration in adopting data analytic activities for online classes is providing data. In the case of a CURE, students may generate the data to analyze. However, in courses where this is not the case, data analytical activities can still be used. NSF‐supported resources, such as QUBES, archive datasets and activities that have successfully been used in courses. Additionally, resources exist for specific data types, such as iDigBio for museum collection data, GenBank for genetic data, HormoneBase for measures of hormones in free‐living vertebrates, and the National Ecological Observatory Network for large ecology data sets (also see Table 2).

## 4 CONCLUSIONS

The 2020 COVID‐19 pandemic brought a sudden transition to online teaching, upending many educational practices and causing considerable stress for both instructors and learners. However, in this manuscript, we reframe this transition from a loss of in‐person instruction to an opportunity to build inclusive digital spaces from the ground up. We have highlighted a number of considerations for faculty, such as culturally competent pedagogy, universal design for learning, and trauma‐informed pedagogy, that can help learners and instructors establish a positive classroom community, even from a distance. We have also highlighted a number of active learning interventions that can be adopted with varying levels of effort.

Although instructors may be unable to address societal inequities in the course of a year, we believe that we can intentionally design our online classroom to draw in students who are often excluded from traditional in‐person classroom participation. This is especially important for Ecology and Evolutionary Biology (EEB) as these fields have traditionally been dominated by white individuals and have lower diversity than other STEM fields (Leslie et al., 2015). From 2008–2018, individuals identifying as white, non‐Hispanic/non‐Latino earned 81.5% and 86.5% of PhDs in evolution and ecology, respectively (NSF survey data; https://ncses.nsf.gov/pubs/nsf19301/data). Given these data, it is not surprising that Black, Latinx, Indigenous and other nonwhite individuals are also underrepresented at the faculty level in EEB (Graves, 2019; O’Brien et al., 2020). Various reasons for these disparities in EEB, and across STEM fields, have been proposed, but racism, sexism, and lack of inclusion are driving factors (Kent et al., 2020; Miriti, 2020; North, 2020; O’Brien et al., 2020; Tseng et al., 2020; Wanelik et al., 2020). The history and narratives of EEB have been primarily shaped by white men as they were the ones who had access to resources and held positions of power, and this holds true across STEM fields (Carter et al., 2019; Lee, 2020; Zuberi & Bonilla‐Silva, 2008).

The first step to inclusive teaching echoes bell hooks’ ideas of self‐actualization (hooks, 1994) and is to develop self‐awareness around one's implicit biases and relationship to established power structures (Asplund & Welle, 2018; Dewsbury & Brame, 2019). Instructors must be willing to devote mental energy to introspection and acknowledge their role in perpetuating oppression in the classroom. This work is uncomfortable and time‐intensive, which may impede widespread implementation of inclusive teaching across institutions (DiAngelo & Sensoy, 2010; Lombardi et al., 2011). Additionally, many science instructors hold the assumption that STEM fields are somehow “unbiased” and immune to societal injustices (Saini, 2020; Smith & Scharmann, 1999; Wheeler et al., 2019), making inclusive teaching challenging. Lastly, traditional STEM curricula tend to promote racist, sexist, and Eurocentric ideas (Black Lives Matter in Ecology & Evolution, 2020; Hayssen, 2020; Hayssen & Orr, 2017; Peters, 2015; Smedley & Smedley, 2005; Vakil & Ayers, 2019). Disparities start early, as a recent study found introductory biology textbooks were more likely to highlight men scientists, and none of the books analyzed highlighted a Black woman scientist (Wood et al., 2020). These biases impact students’ first view of the field and can shape the ideas of who belongs in science; these disparities are something instructors can actively address in their individual syllabi and curriculum.

We acknowledge that our recommendations alone will not create an equitable, inclusive, and socially just learning experience for our students. The pandemic has highlighted the broad systemic inequities in higher education, and it will take much more than active learning strategies or mindful attention to course design to address the ongoing issues of who we center and who we exclude from education. But we believe that this paper can serve as an entry point and we hope it will inspire instructors to start the long journey of personal and pedagogical transformation. We encourage readers to think critically about their own courses and syllabi, and to make a pledge to change at least one thing to increase inclusion in their classroom. We challenge readers to truly reflect on their own views and behaviors as well as those of their department and institution. We also challenge readers to critically examine what is meant by inclusive teaching, in their own mind, and in their institution and classrooms. Lastly, we hope readers will determine actionable steps to increase inclusion going forward.

## Glossary

● ***Active Learning*** : Active learning refers to educational processes in which students are not passive participants in learning, rather they are actively engaged in their own learning. On one end of the spectrum, this might mean simple activities such as minute papers, where students write a short reflection. On the opposite end, this might include course‐based research, where students spend most of their time researching.
● ***Scientific teaching*** : Scientific teaching refers to using the scientific process to understand pedagogy. An instructor using scientific teaching incorporates methods, such as active learning, that have been shown effective for communicating material and including more students.
● ***Backwards design*** : Backwards design refers to writing learning outcomes before starting to design activities, lectures, and assessments. This technique can help the instructor design a cohesive course that successfully meets predetermined learning outcomes.
● ***Inclusive Teaching:*** Inclusive teaching refers to teaching in which instructors understand and respond to dynamics both inside and outside the classroom that shape learners’ classroom experience and ability to learn. The next three terms in this glossary are all components of inclusive teaching.
● ***Culturally Competent Teaching*** : Culturally competent teaching refers to teaching with an ability to successfully teach students from backgrounds other than the instructor's own. Culturally competent instructors will be able to understand factors in the learners’ cultural background that may impact their ability to learn.
● ***Universal Design for Learning (UDL)*** : This is adapted from universal design, a technological concept that includes closed‐captioning and accessibility features. Universal design for learning is the idea of taking these same concepts to apply in the classroom. Examples of this could include closed‐captioning video lectures and providing learning objectives so that students can self‐assess their learning.
● ***Trauma‐informed pedagogy*** : Trauma‐informed pedagogy refers to the practice of understanding the influence of external, traumatic events on student learning. For example, in the 2020 school year, US college students learned during a pandemic and, depending on when the school year ended, civil unrest following widespread racist violence. Trauma‐informed pedagogy means understanding how these events have disrupted the lives of learners and finding ways to accommodate students.
● ***Face‐to‐face learning*** : Face‐to‐face classes are those in which the learners and instructor meet together to deliver course materials in person.
● ***Synchronous online learning*** : Synchronous teaching, broadly, refers to a situation in which the instructor and the learners are logged into a platform to conduct lecture or activities together at an appointed time.
● ***Asynchronous online learning*** : Asynchronous learning refers to a situation where students and faculty do not log on to a platform at the same time. This could mean students view lectures and complete activities on their own schedule, or could have a more structured system of check‐ins.
● ***Hybrid online learning*** : Hybrid online learning has some online component to the class, but the entire course is not wholly online. An example could be viewing lecture digitally, but meeting for a problem‐solving activity.
● ***HyFlex online learning*** : HyFlex typically involves having some attendees in the classroom, and some participants online. In HyFlex learning, the online attendees could be determined beforehand, or who is online and who is in‐person could change throughout the semester.
● ***Flipped course*** : Flipped classrooms refer to classrooms in which students use classroom materials outside class time. This may refer to watching a video lecture prior to class or performing a reading. Class time is then typically used to do activities or assessments. Hybrid learning classrooms are often implemented as flipped classrooms.
● ***Cloud‐based platform*:** These are applications, services, or resources that are stored on a remote server and are available to users via an Internet connection. Users can thus access tools freely from a remote location as long as they have Internet access.

## CONFLICTS OF INTEREST

None.

## AUTHOR CONTRIBUTIONS

**Breanna N. Harris:** Conceptualization (lead); Project administration (lead); Resources (equal); Visualization (equal); Writing‐original draft (lead); Writing‐review & editing (equal). **Pumtiwitt C. McCarthy:** Conceptualization (equal); Resources (equal); Writing‐original draft (equal); Writing‐review & editing (equal). **April M. Wright:** Conceptualization (equal); Resources (equal); Writing‐original draft (equal); Writing‐review & editing (supporting). **Heidi Schutz:** Conceptualization (equal); Resources (equal); Writing‐original draft (equal); Writing‐review & editing (equal). **Kate S. Boersma:** Conceptualization (equal); Resources (equal); Writing‐original draft (equal); Writing‐review & editing (equal). **Stephanie L. Shepherd:** Conceptualization (equal); Resources (lead); Visualization (equal); Writing‐original draft (equal); Writing‐review & editing (supporting). **Lathiena A. Manning:** Conceptualization (equal); Resources (equal); Writing‐original draft (equal); Writing‐review & editing (supporting). **Jessica L. Malisch:** Conceptualization (equal); Writing‐original draft (equal); Writing‐review & editing (supporting). **Roni M. Ellington:** Conceptualization (equal); Writing‐original draft (supporting).

## Supporting information

Appendix S1

## Data Availability

This manuscript was prepared for the special issue for taking learning online. The authors did not generate or analyze and data for preparation of this manuscript.

## References

[ece36915-bib-0001] AAAS (2011). Vision and change in undergraduate biology education: A call to action. Washington, DC: American Association for the Advancement of Science.

[ece36915-bib-0002] AAAS (2015). Vision and change in undergraduate biology education: Chronicling change, inspiring the future. Washington, DC: American Association for the Advancement of Science.

[ece36915-bib-0003] AAAS (2018). Vision and Change in undergraduate biology education: Unpacking a moment and sharing lessons learned. Washington, DC: American Association for the Advancement of Science.

[ece36915-bib-0004] Adams, J. P. , Brissenden, G. , Lindell, R. S. , Slater, T. F. , & Wallace, J. (2002). Observations of student behavior in collaborative learning groups. Astronomy Education Review, 1(1), 25–32.

[ece36915-bib-0005] Aguillon, S. M. , Siegmund, G.‐F. , Petipas, R. H. , Drake, A. G. , Cotner, S. , & Ballen, C. J. (2020). Gender differences in student participation in an active‐learning classroom. Cbe—life Sciences Education, 19(2), ar12 10.1187/cbe.19-03-0048 32453677 PMC8697656

[ece36915-bib-0006] Ainscow, M. , Booth, T. , & Dyson, A. (2006). Improving schools, developing inclusion. Routledge.

[ece36915-bib-0007] An, D. , & Carr, M. (2017). Learning styles theory fails to explain learning and achievement: Recommendations for alternative approaches. Personality and Individual Differences, 116, 410–416.

[ece36915-bib-0008] Anderson, L. W. , Krathwohl, D. R. , Airasian, P. W. , Cruikshank, K. A. , Mayer, R. E. , Pintrich, P. R. , Raths, J. , & Wittrock, M. C. (2001). A taxonomy for learning, teaching, and assessing: A revision of Bloom’s Taxonomy of Educational Objectives (Complete edition). Longman.

[ece36915-bib-0009] Andrews, T. M. , Leonard, M. J. , Colgrove, C. A. , & Kalinowski, S. T. (2011). Active learning not associated with student learning in a random sample of college biology courses. Cbe—life Sciences Education, 10(4), 394–405. 22135373 10.1187/cbe.11-07-0061 PMC3228657

[ece36915-bib-0010] Asplund, M. , & Welle, C. G. (2018). Advancing science: How bias holds us back. Neuron, 99(4), 635–639. 10.1016/j.neuron.2018.07.045 30138587

[ece36915-bib-0011] Aucejo, E. M. , French, J. F. , Araya, M. P. U. , & Zafar, B. (2020). The Impact of COVID‐19 on Student Experiences and Expectations: Evidence from a Survey. National Bureau of Economic Research. 10.1016/j.jpubeco.2020.104271 PMC7451187 32873994

[ece36915-bib-0012] Auchincloss, L. C. , Laursen, S. L. , Branchaw, J. L. , Eagan, K. , Graham, M. , Hanauer, D. I. , Lawrie, G. , McLinn, C. M. , Pelaez, N. , Rowland, S. , Towns, M. , Trautmann, N. M. , Varma‐Nelson, P. , Weston, T. J. , & Dolan, E. L. (2014). Assessment of course‐based undergraduate research experiences: A meeting report. CBE Life Sciences Education, 13(1), 29–40. 10.1187/cbe.14-01-0004 24591501 PMC3940459

[ece36915-bib-0013] Auster, E. R. , & Wylie, K. K. (2006). Creating active learning in the classroom: A systematic approach. Journal of Management Education, 30(2), 333–353. 10.1177/1052562905283346

[ece36915-bib-0014] Balgopal, M. M. , Casper, A. M. A. , Wallace, A. M. , Laybourn, P. J. , & Brisch, E. (2018). Writing matters: Writing‐to‐learn activities increase undergraduate performance in cell biology. BioScience, 68(6), 445–454. 10.1093/biosci/biy042

[ece36915-bib-0015] Ballen, C. J. (2020). Enhancing diversity in college science with active learning In Mintzes J. & Walter E. (Eds.), Active Learning in College Science (pp. 873–887). Springer 10.1007/978-3-030-33600-4_54

[ece36915-bib-0016] Ballen, C. J. , Blum, J. E. , Brownell, S. , Hebert, S. , Hewlett, J. , Klein, J. R. , McDonald, E. A. , Monti, D. L. , Nold, S. C. , Slemmons, K. E. , Soneral, P. A. G. , & Cotner, S. (2017). A call to develop course‐based undergraduate research experiences (CUREs) for Nonmajors courses. CBE Life Sciences Education, 16(2). 10.1187/cbe.16-12-0352 PMC5459265 28450449

[ece36915-bib-0017] Ballen, C. J. , Wieman, C. , Salehi, S. , Searle, J. B. , & Zamudio, K. R. (2017). Enhancing diversity in undergraduate science: Self‐efficacy drives performance gains with active learning. Cbe—life Sciences Education, 16(4), ar56 10.1187/cbe.16-12-0344 29054921 PMC5749958

[ece36915-bib-0018] Bangera, G. , & Brownell, S. E. (2014). Course‐based undergraduate research experiences can make scientific research more inclusive. Cbe—life Sciences Education, 13(4), 602–606. 10.1187/cbe.14-06-0099 25452483 PMC4255347

[ece36915-bib-0019] Bean, J. C. (2011). Engaging ideas: The professor’s guide to integrating writing, critical thinking, and active learning in the classroom. John Wiley & Sons.

[ece36915-bib-0020] Black Lives Matter in ecology and evolution (2020). Nat Ecol Evol, 4, 893–894. 10.1038/s41559-020-1250-2 32576974

[ece36915-bib-0021] Bloom, B. S. (1956). Taxonomy of educational objectives. Vol. 1: Cognitive domain. McKay, 20, 24.

[ece36915-bib-0022] Branchaw, J. L. , Pape‐Lindstrom, P. A. , Tanner, K. D. , Bissonnette, S. A. , Cary, T. L. , Couch, B. A. , Crowe, A. J. , Knight, J. K. , Semsar, K. , & Smith, J. I. (2020). Resources for teaching and assessing the vision and change biology core concepts. Cbe—life Sciences Education, 19(2), es1. 32357095 10.1187/cbe.19-11-0243 PMC8697664

[ece36915-bib-0023] Brownell, S. E. , Freeman, S. , Wenderoth, M. P. , & Crowe, A. J. (2014). BioCore guide: A tool for interpreting the core concepts of vision and change for biology majors. CBE‐Life Sciences Education, 13(2), 200–211. 10.1187/cbe.13-12-0233 26086653 PMC4041499

[ece36915-bib-0024] Brownell, S. E. , & Tanner, K. D. (2012). Barriers to faculty pedagogical change: Lack of training, time, incentives, and… tensions with professional identity?. Cbe—life Sciences Education, 11(4), 339–346. 10.1187/cbe.12-09-0163 23222828 PMC3516788

[ece36915-bib-0025] Burrow, A. K. (2018). Teaching introductory ecology with problem‐based learning. The Bulletin of the Ecological Society of America, 99(1), 137–150. 10.1002/bes2.1364

[ece36915-bib-0026] Camfield, E. K. , Beaster‐Jones, L. , Miller, A. D. , & Land, K. M. (2020). Using writing in science class to understand and activate student engagement and self‐efficacy In Mintzes J. & Walter E. (Eds.), Active Learning in College Science (pp. 89–105). Springer.

[ece36915-bib-0027] Camfield, E. K. , & Land, K. M. (2017). The evolution of student engagement: Writing improves teaching in introductory biology courses. Bioscene: Journal of College Biology Teaching, 43(1), 20–26.

[ece36915-bib-0028] Campbell, L. O. , Heller, S. , & Pulse, L. (2020). Student‐created video: An active learning approach in online environments. Interactive Learning Environments, 1–10. 10.1080/10494820.2020.1711777

[ece36915-bib-0029] Canning, E. A. , Muenks, K. , Green, D. J. , & Murphy, M. C. (2019). STEM faculty who believe ability is fixed have larger racial achievement gaps and inspire less student motivation in their classes. *Science* . Advances, 5(2), eaau4734 10.1126/sciadv.aau4734 PMC6377274 30793027

[ece36915-bib-0030] Carter, D. F. , Dueñas, J. E. R. , & Mendoza, R. (2019). Critical examination of the role of STEM in propagating and maintaining race and gender disparities In Paulsen M. & Perna L. (Eds.), Higher education: Handbook of theory and research (pp. 39–97). Springer.

[ece36915-bib-0031] Cary, T. , & Branchaw, J. (2017). Conceptual elements: A detailed framework to support and assess student learning of biology core concepts. Cbe—life Sciences Education, 16(2), ar24 10.1187/cbe.16-10-0300 28450444 PMC5459242

[ece36915-bib-0032] Cavanagh, A. J. , Aragón, O. R. , Chen, X. , Couch, B. A. , Durham, M. F. , Bobrownicki, A. , Hanauer, D. I. , & Graham, M. J. (2016). Student buy‐in to active learning in a college science course. Cbe—life Sciences Education, 15(4), ar76 10.1187/cbe.16-07-0212 27909026 PMC5132373

[ece36915-bib-0033] Cavanagh, A. J. , Chen, X. , Bathgate, M. , Frederick, J. , Hanauer, D. I. , & Graham, M. J. (2018). Trust, growth mindset, and student commitment to active learning in a college science course. Cbe—life Sciences Education, 17(1), ar10 10.1187/cbe.17-06-0107 29378750 PMC6007784

[ece36915-bib-0034] Chapman, K. J. , Meuter, M. , Toy, D. , & Wright, L. (2006). Can’t we pick our own groups? The influence of group selection method on group dynamics and outcomes. Journal of Management Education, 30(4), 557–569. 10.1177/1052562905284872

[ece36915-bib-0035] Chávez, K. , & Mitchell, K. M. W. (2020). Exploring bias in student evaluations: Gender, race, and ethnicity. PS: Political Science & Politics, 53(2), 270–274.

[ece36915-bib-0036] Chen, P. , Chavez, O. , Ong, D. C. , & Gunderson, B. (2017). Strategic resource use for learning: A self‐administered intervention that guides self‐reflection on effective resource use enhances academic performance. Psychological Science, 28(6), 774–785. 10.1177/0956797617696456 28447894

[ece36915-bib-0037] Choe, R. C. , Scuric, Z. , Eshkol, E. , Cruser, S. , Arndt, A. , Cox, R. , Toma, S. P. , Shapiro, C. , Levis‐Fitzgerald, M. , Barnes, G. , & Crosbie, R. H. (2019). Student Satisfaction and Learning Outcomes in Asynchronous Online Lecture Videos. Cbe—life Sciences Education, 18(4), ar55 10.1187/cbe.18-08-0171 31675279 PMC6829069

[ece36915-bib-0038] Chou, S. , & Liu, C. (2005). Learning effectiveness in a Web‐based virtual learning environment: A learner control perspective. Journal of Computer Assisted Learning, 21(1), 65–76. 10.1111/j.1365-2729.2005.00114.x

[ece36915-bib-0039] Clemmons, A. , Timbrook, J. , Herron, J. , & Crowe, A. (2020). BioSkills guide: Development and national validation of a tool for interpreting the vision and change core competencies. *BioRxiv*, 10.1101/2020.01.11.902882 PMC8693931 33001766

[ece36915-bib-0040] Cooper, K. M. , Soneral, P. A. G. , & Brownell, S. E. (2017). Define your goals before you design a CURE: A call to use backward design in planning course‐based undergraduate research experiences. Journal of Microbiology & Biology Education, 18(2), 18.2.30 10.1128/jmbe.v18i2.1287 PMC5440170 28656069

[ece36915-bib-0041] Couch, B. A. , Wright, C. D. , Freeman, S. , Knight, J. K. , Semsar, K. , Smith, M. K. , Summers, M. M. , Zheng, Y. , Crowe, A. J. , & Brownell, S. E. (2019). GenBio‐MAPS: A programmatic assessment to measure student understanding of Vision and change core concepts across general biology programs. Cbe—life Sciences Education, 18(1), ar1. 30681904 10.1187/cbe.18-07-0117 PMC6757222

[ece36915-bib-0042] Crews, T. , & Butterfield, J. (2014). Data for flipped classroom design: Using student feedback to identify the best components from online and face‐to‐face classes. Higher Education Studies, 4(3), 38–47. 10.5539/hes.v4n3p38

[ece36915-bib-0043] Crowe, A. , Dirks, C. , & Wenderoth, M. P. (2008). Biology in bloom: Implementing Bloom’s taxonomy to enhance student learning in biology. Cbe—life Sciences Education, 7(4), 368–381. 10.1187/cbe.08-05-0024 19047424 PMC2592046

[ece36915-bib-0044] Dahlstrom‐Hakki, I. , Alstad, Z. , & Banerjee, M. (2020). Comparing synchronous and asynchronous online discussions for students with disabilities: The impact of social presence. Computers & Education, 150, 103842 10.1016/j.compedu.2020.103842

[ece36915-bib-0045] Dallimore, E. J. , Hertenstein, J. H. , & Platt, M. B. (2013). Impact of cold‐calling on student voluntary participation. Journal of Management Education, 37(3), 305–341. 10.1177/1052562912446067

[ece36915-bib-0046] Darby, F. (2020). 5 Ways to Connect With Online Students. *The Chronicle of Higher Education* Retrieved from https://www.chronicle.com/article/5‐Ways‐to‐Connect‐With‐Online/249077

[ece36915-bib-0047] Darby, F. , & Lang, J. M. (2019). Small teaching online: Applying learning science in online classes. John Wiley & Sons.

[ece36915-bib-0048] Dell, C. A. , Dell, T. F. , & Blackwell, T. L. (2015). Applying universal design for learning in online courses: Pedagogical and practical considerations. Journal of Educators Online, 12(2), 166–192. 10.9743/JEO.2015.2.1

[ece36915-bib-0049] DeLozier, S. J. , & Rhodes, M. G. (2017). Flipped classrooms: A review of key ideas and recommendations for practice. Educational Psychology Review, 29(1), 141–151. 10.1007/s10648-015-9356-9

[ece36915-bib-0050] Deslauriers, L. , McCarty, L. S. , Miller, K. , Callaghan, K. , & Kestin, G. (2019). Measuring actual learning versus feeling of learning in response to being actively engaged in the classroom. Proceedings of the National Academy of Sciences of the United States of America, 116(39), 19251–19257. 10.1073/pnas.1821936116 31484770 PMC6765278

[ece36915-bib-0051] Dewsbury, B. M. (2017). On faculty development of STEM inclusive teaching practices. FEMS Microbiology Letters, 364(18). 10.1093/femsle/fnx179 28922842

[ece36915-bib-0052] Dewsbury, B. M. (2020). Deep teaching in a college STEM classroom. Cultural Studies of Science Education, 15(1), 169–191. 10.1007/s11422-018-9891-z

[ece36915-bib-0053] Dewsbury, B. , & Brame, C. J. (2019). Inclusive teaching. Cbe—life Sciences Education, 18(2), fe2 10.1187/cbe.19-01-0021 31025917 PMC7058128

[ece36915-bib-0054] DiAngelo, R. , & Sensoy, Ö. (2010). “OK, I get it! Now tell me how to do it!”: Why we can’t just tell you how to do critical multicultural education. Multicultural Perspectives, 12(2), 97–102. 10.1080/15210960.2010.481199

[ece36915-bib-0055] Dutcher, E. G. , & Rodet, C. S. (2018). Which two heads are better than one?: Uncovering the positive effects of diversity in creative teams. SSRN. 10.2139/ssrn.3283355

[ece36915-bib-0056] Dweck, C. (2015). Carol Dweck revisits the growth mindset. Education Week, 35(5), 20–24.

[ece36915-bib-0057] Eddy, S. L. , Brownell, S. E. , & Wenderoth, M. P. (2014). Gender gaps in achievement and participation in multiple introductory biology classrooms. Cbe—life Sciences Education, 13(3), 478–492. 10.1187/cbe.13-10-0204 25185231 PMC4152209

[ece36915-bib-0058] Estrada, M. , Burnett, M. , Campbell, A. G. , Campbell, P. B. , Denetclaw, W. F. , Gutiérrez, C. G. , Hurtado, S. , John, G. H. , Matsui, J. , McGee, R. , Okpodu, C. M. , Robinson, T. J. , Summers, M. F. , Werner‐Washburne, M. , & Zavala, M. E. (2016). Improving underrepresented minority student persistence in STEM. Cbe—life Sciences Education, 15(3), es5 10.1187/cbe.16-01-0038 27543633 PMC5008901

[ece36915-bib-0059] Favero, T. G. , & Van Hoomissen, J. D. (2019). Leveraging undergraduate research to identify culturally relevant examples in the anatomy and physiology curriculum. Advances in Physiology Education, 43(4), 561–566. 10.1152/advan.00023.2019 31697165

[ece36915-bib-0060] Finelli, C. J. , & Borrego, M. (2020). Evidence‐based strategies to reduce student resistance to active learning In Mintzes J. & Walter E. (Eds.), Active learning in college science, (pp. 943–952). Springer.

[ece36915-bib-0061] Forlin, C. , & Loreman, T. (2014). Measuring inclusive education. Emerald Group Publishing.

[ece36915-bib-0062] Frasier, T. R. , & Roderick, C. (2011). Improving how evolution is taught: Facilitating a shift from memorization to evolutionary thinking. Evolution: Education and Outreach, 4(2), 298–307.

[ece36915-bib-0063] Freeman, R. B. , & Huang, W. (2015). Collaborating with people like me: Ethnic coauthorship within the United States. Journal of Labor Economics, 33(S1), S289–S318. 10.1086/678973

[ece36915-bib-0064] Freeman, S. , Eddy, S. L. , McDonough, M. , Smith, M. K. , Okoroafor, N. , Jordt, H. , & Wenderoth, M. P. (2014). Active learning increases student performance in science, engineering, and mathematics. Proceedings of the National Academy of Sciences of the United States of America, 111(23), 8410–8415. 10.1073/pnas.1319030111 24821756 PMC4060654

[ece36915-bib-0065] Freeman, S. , O’Connor, E. , Parks, J. W. , Cunningham, M. , Hurley, D. , Haak, D. , Dirks, C. , & Wenderoth, M. P. (2007). Prescribed active learning increases performance in introductory biology. Cbe—life Sciences Education, 6(2), 132–139. 10.1187/cbe.06-09-0194 17548875 PMC1885904

[ece36915-bib-0066] Freeman, S. , Theobald, R. , Crowe, A. J. , & Wenderoth, M. P. (2017). Likes attract: Students self‐sort in a classroom by gender, demography, and academic characteristics. Active Learning in Higher Education, 18(2), 115–126. 10.1177/1469787417707614

[ece36915-bib-0067] Friedrich, K. A. , Sellers, S. L. , & Burstyn, J. N. (2008). 9: Thawing the Chilly Climate: Inclusive Teaching Resources for Science, Technology, Engineering, and Math In Roberston D., & Nilson L. (Eds.), To improve the academy: Resources for faculty, instructional, and organizational development (pp. 133–141). Jossey‐Bass.

[ece36915-bib-0068] Garrison, D. R. , Anderson, T. , & Archer, W. (1999). Critical inquiry in a text‐based environment: Computer conferencing in higher education. The Internet and Higher Education, 2(2–3), 87–105. 10.1016/S1096-7516(00)00016-6

[ece36915-bib-0069] Graves, J. L. (2019). African Americans in evolutionary science: Where we have been, and what’s next. Evolution: Education and Outreach, 12(1), 18.

[ece36915-bib-0070] Haak, D. C. , HilleRisLambers, J. , Pitre, E. , & Freeman, S. (2011). Increased structure and active learning reduce the achievement gap in introductory biology. Science, 332(6034), 1213–1216. 21636776 10.1126/science.1204820

[ece36915-bib-0071] Hackl, E. , & Ermolina, I. (2019). Inclusion by design: Embedding inclusive teaching practice into design and preparation of laboratory classes. Currents in Pharmacy Teaching and Learning, 11(12), 1323–1334. 10.1016/j.cptl.2019.09.012 31836160

[ece36915-bib-0072] Handelsman, J. , Miller, S. , & Pfund, C. (2007). Scientific teaching. Macmillan. 10.1126/science.1166032 19039122

[ece36915-bib-0073] Harris, R. B. , Mack, M. R. , Bryant, J. , Theobald, E. J. , & Freeman, S. (2020). Reducing achievement gaps in undergraduate general chemistry could lift underrepresented students into a “hyperpersistent zone”. Science Advances, 6(24), eaaz5687. 32577510 10.1126/sciadv.aaz5687 PMC7286681

[ece36915-bib-0074] Hayssen, V. (2020). Misconceptions about conception and other fallacies: Historical bias in reproductive biology. Integrative and Comparative Biology, 60(3), 683–691. 10.1093/icb/icaa035 32396629

[ece36915-bib-0075] Hayssen, V. , & Orr, T. J. (2017). Reproduction in mammals: The female perspective. JHU Press.

[ece36915-bib-0076] Herreid, C. F. , Prud’homme‐Genereux, A. , Schiller, N. A. , Herreid, K. F. , & Wright, C. (2016). What makes a good case, revisited: The survey monkey tells all. Journal of College Science Teaching, 46(1), 60.

[ece36915-bib-0077] Holt, E. A. (2012). Education improves plagiarism detection by biology undergraduates. BioScience, 62(6), 585–592. 10.1525/bio.2012.62.6.9

[ece36915-bib-0078] Holt, E. A. , Fagerheim, B. , & Durham, S. (2014). Online plagiarism training falls short in biology classrooms. Cbe—life Sciences Education, 13(1), 83–89. 10.1187/cbe.13-08-0146 24591507 PMC3940467

[ece36915-bib-0079] Hood, S. , Barrickman, N. , Djerdjian, N. , Farr, M. , Gerrits, R. J. , Lawford, H. , Magner, S. , Ott, B. , Ross, K. , & Roychowdury, H. (2020). Some believe, not all achieve: the role of active learning practices in anxiety and academic self‐efficacy in first‐generation college students. Journal of Microbiology & Biology Education, 21(1).21.1.19 10.1128/jmbe.v21i1.2075 PMC7148146 32313594

[ece36915-bib-0080] Hooks, B. (1994). Teaching to transgress. Education as a freedom of practice. Routledge.

[ece36915-bib-0081] Howard, T. C. (2003). Culturally relevant pedagogy: Ingredients for critical teacher reflection. Theory into Practice, 42(3), 195–202. 10.1207/s15430421tip4203_5

[ece36915-bib-0082] Hughes‐Darden, C. , Ellington, R. M. , Zaveri, J. , Bapna, S. , Akli, L. , Hargett, S. , Bhattacharya, P. , Emdad, A. , & Nkwanta, A. (2019). Interventions addressing recruitment and retention of underrepresented minority groups in undergraduate STEM disciplines In Mack K. M., Winter K., & Soto M. (Eds.), Culturally responsive strategies for reforming STEM higher education (pp. 229–247). Emerald Group Publishing.

[ece36915-bib-0083] Hulleman, C. S. , & Harackiewicz, J. M. (2009). Promoting interest and performance in high school science classes. Science, 326(5958), 1410–1412. 19965759 10.1126/science.1177067

[ece36915-bib-0084] Jang, S. (2017). Cultural brokerage and creative performance in multicultural teams. Organization Science, 28(6), 993–1009. 10.1287/orsc.2017.1162

[ece36915-bib-0085] Kalinowski, S. T. , Leonard, M. J. , & Andrews, T. M. (2010). Nothing in evolution makes sense except in the light of DNA. Cbe—life Sciences Education, 9(2), 87–97. 10.1187/cbe.09-12-0088 20516354 PMC2879385

[ece36915-bib-0086] Kent, S. R. , John, J. E. , & Robnett, R. D. (2020). “Maybe these fields just don’t interest them:” Gender and ethnic differences in attributions about STEM inequities. International Journal of Gender, Science and Technology, 12(1), 97–121.

[ece36915-bib-0087] Killpack, T. L. , & Melón, L. C. (2016). Toward inclusive STEM classrooms: What personal role do faculty play?. Cbe—life Sciences Education, 15(3), es3 10.1187/cbe.16-01-0020 27496362 PMC5008899

[ece36915-bib-0088] Kilpatrick, A. M. , Anjum, A. , & Welch, L. (2020). Ten simple rules for designing learning experiences that involve enhancing computational biology Wikipedia articles. PLOS Computational Biology, 16(5), e1007868 10.1371/journal.pcbi.1007868 32407308 PMC7224448

[ece36915-bib-0089] Kim, Y. K. , & Sax, L. J. (2009). Student–faculty interaction in research universities: Differences by student gender, race, social class, and first‐generation status. Research in Higher Education, 50(5), 437–459. 10.1007/s11162-009-9127-x

[ece36915-bib-0090] Knight, J. K. , Wise, S. B. , & Sieke, S. (2016). Group random call can positively affect student in‐class clicker discussions. Cbe—life Sciences Education, 15(4), ar56 10.1187/cbe.16-02-0109 27856544 PMC5132353

[ece36915-bib-0091] Konieczny, P. (2016). Teaching with Wikipedia in a 21st‐century classroom: Perceptions of Wikipedia and its educational benefits. Journal of the Association for Information Science and Technology, 67(7), 1523–1534.

[ece36915-bib-0092] Kuh, G. D. (2008). High‐impact educational practices: What they are, who has access to them, and why they matter. Association of American Colleges and Universities Press.

[ece36915-bib-0093] Lacey, M. M. , Campbell, S. G. , Shaw, H. , & Smith, D. P. (2020). Self‐selecting peer groups formed within the laboratory environment have a lasting effect on individual student attainment and working practices. FEBS Open Bio, 10(7), 1194–1209. 10.1002/2211-5463.12902 PMC7327925 32438509

[ece36915-bib-0094] Ladson‐Billings, G. (1992). Reading between the lines and beyond the pages: A culturally relevant approach to literacy teaching. Theory into Practice, 31(4), 312–320. 10.1080/00405849209543558

[ece36915-bib-0095] Ladson‐Billings, G. (1995). Toward a theory of culturally relevant pedagogy. American Educational Research Journal, 32(3), 465–491. 10.3102/00028312032003465

[ece36915-bib-0096] Ladson‐Billings, G. (2014). Culturally relevant pedagogy 2.0: Aka the remix. Harvard Educational Review, 84(1), 74–84.

[ece36915-bib-0097] Lang, J. M. (2016). Small teaching: Everyday lessons from the science of learning. John Wiley & Sons. 10.1097/JPA.0000000000000330 33953147

[ece36915-bib-0098] Lawrie, G. , Marquis, E. , Fuller, E. , Newman, T. , Qiu, M. , Nomikoudis, M. , Roelofs, F. , & Van Dam, L. (2017). Moving towards inclusive learning and teaching: A synthesis of recent literature. Teaching & Learning Inquiry, 5(1), 1–13. 10.20343/teachlearninqu.5.1.3

[ece36915-bib-0099] Lee, D. N. (2020). Diversity and inclusion activisms in animal behaviour and the ABS: A historical view from the USA. Animal Behaviour, 164, 273–280. 10.1016/j.anbehav.2020.03.019

[ece36915-bib-0100] Lerner, J. , & Lomi, A. (2018). Diverse teams tend to do good work in Wikipedia (but jacks of all trades don’t). 2018 IEEE/ACM International Conference on Advances in Social Networks Analysis and Mining (ASONAM), 214–221.

[ece36915-bib-0101] Lerner, S. , Magrane, D. , & Friedman, E. (2009). Teaching teamwork in medical education. Mount Sinai Journal of Medicine: A Journal of Translational and Personalized Medicine: A Journal of Translational and Personalized Medicine, 76(4), 318–329. 10.1002/msj.20129 19642146

[ece36915-bib-0102] Leslie, S.‐J. , Cimpian, A. , Meyer, M. , & Freeland, E. (2015). Expectations of brilliance underlie gender distributions across academic disciplines. Science, 347(6219), 262–265. 25593183 10.1126/science.1261375

[ece36915-bib-0103] Livingstone, D. , & Lynch, K. (2002). Group project work and student‐centred active learning: Two different experiences. Journal of Geography in Higher Education, 26(2), 217–237. 10.1080/03098260220144748

[ece36915-bib-0104] Lombardi, A. R. , Murray, C. , & Gerdes, H. (2011). College faculty and inclusive instruction: Self‐reported attitudes and actions pertaining to Universal Design. Journal of Diversity in Higher Education, 4(4), 250 10.1037/a0024961

[ece36915-bib-0105] Mack, K. (2019). At high tide: Teaching to increase diversity and equity in STEM. AAC&U News. Retrieved from https://www.aacu.org/aacu‐news/newsletter/high‐tide‐teaching‐increase‐diversity‐and‐equity‐stem

[ece36915-bib-0106] MacNell, L. , Driscoll, A. , & Hunt, A. N. (2015). What’s in a name: Exposing gender bias in student ratings of teaching. Innovative Higher Education, 40(4), 291–303. 10.1007/s10755-014-9313-4

[ece36915-bib-0107] Malisch, J. L. , Harris, B. N. , Sherrer, S. M. , Lewis, K. A. , Shepherd, S. L. , McCarthy, P. C. , Spott, J. L. , Karam, E. P. , Moustaid‐Moussa, N. , Calarco, J. M. , Ramalingam, L. , Talley, E. T. , Canas‐Carrell, J. E. , Ardon‐Dryer, K. , Weiser, D. A. , Bernal, X. E. , & Deitloff, J. (2020). Opinion: In the wake of COVID‐19, academia needs new solutions to ensure gender equity. Proceedings of the National Academy of Sciences of the United States of America, 117(27), 15378–15381. 10.1073/pnas.2010636117 32554503 PMC7354923

[ece36915-bib-0108] Matsushita, K. (2018). An invitation to deep active learning In Matsushita K. (Eds.), Deep active learning (pp. 15–33). Springer.

[ece36915-bib-0109] Matta, V. , Luce, T. , & Ciavarro, G. (2010). Exploring impact of self‐selected student teams and academic potential on satisfaction. Information Systems Educators Conference, 27, 1–10.

[ece36915-bib-0110] McGuire, S. Y. , & McGuire, S. (2015). Teach students how to learn: Strategies you can incorporate into any course to improve student metacognition, study skills, and motivation. Stylus Publishing LLC.

[ece36915-bib-0111] McInerney, M. , & McKlindon, A. (2014). Unlocking the door to learning: Trauma‐informed classrooms & transformational schools. Education Law Center (pp. 1–24).

[ece36915-bib-0112] McMurtrie, B. (2019). Many professors want to change their teaching but don’t. One university found out why. *The Chronicle of Higher Education* Retrieved from https://www.chronicle.com/article/Many‐Professors‐Want‐to‐Change/245945

[ece36915-bib-0113] Menking, A. , Erickson, I. , & Pratt, W. (2019). People who can take it: How women Wikipedians negotiate and navigate safety. Proceedings of the 2019 CHI Conference on Human Factors in Computing Systems (pp. 1–14).

[ece36915-bib-0114] Micari, M. , Van Winkle, Z. , & Pazos, P. (2016). Among friends: The role of academic‐preparedness diversity in individual performance within a small‐group STEM learning environment. International Journal of Science Education, 38(12), 1904–1922. 10.1080/09500693.2016.1218091

[ece36915-bib-0115] Michael, J. (2006). Where’s the evidence that active learning works? Advances in Physiology Education, 30, 159–167. 10.1152/advan.00053.2006 17108243

[ece36915-bib-0116] Miles, S. , & Singal, N. (2010). The Education for All and inclusive education debate: Conflict, contradiction or opportunity? International Journal of Inclusive Education, 14(1), 1–15. 10.1080/13603110802265125

[ece36915-bib-0117] Miriti, M. N. (2019). Nature in the eye of the beholder: A case study for cultural humility as a strategy to broaden participation in STEM. Education Sciences, 9(4), 291 10.3390/educsci9040291

[ece36915-bib-0118] Miriti, M. N. (2020). The elephant in the room: Race and STEM diversity. BioScience, 70(3), 237–242. 10.1093/biosci/biz167

[ece36915-bib-0119] Mitchell, K. M. W. , & Martin, J. (2018). Gender bias in student evaluations. PS: Political Science & Politics, 51(3), 648–652.

[ece36915-bib-0120] Montez, N. (2017). Decolonizing Wikipedia through advocacy and activism: The Latina/o theatre Wikiturgy project. Theatre Topics, 27(1), E‐1–E‐9. 10.1353/tt.2017.0012

[ece36915-bib-0121] Moog, R. S. , Spencer, J. N. , & Straumanis, A. R. (2006). Process‐oriented guided inquiry learning: POGIL and the POGIL project. Metropolitan Universities, 17(4), 41–52.

[ece36915-bib-0122] Moore, J. A. (1984). Science as a way of knowing—Evolutionary biology. American Zoologist, 24(2), 467–534. 10.1093/icb/24.2.467

[ece36915-bib-0123] Morrell, C. , & Parker, C. (2013). Adjusting micromessages to improve equity in STEM. Diversity & Democracy, 16(2).

[ece36915-bib-0124] Morrison, K. A. , Robbins, H. H. , & Rose, D. G. (2008). Operationalizing culturally relevant pedagogy: A synthesis of classroom‐based research. Equity & Excellence in Education, 41(4), 433–452. 10.1080/10665680802400006

[ece36915-bib-0125] Nahar, K. , & Chowdhury, R. (2019). Effectiveness of flipped classroom model in distance learning. Proceedings of the 30th Annual Conference for the Australasian Association for Engineering Education (AAEE 2019).

[ece36915-bib-0126] Newhouse, K. (2020). Four core priorities for trauma‐informed distance learning. KQED Mindshift. Retrieved from https://www.kqed.org/mindshift/55679/four‐core‐priorities‐for‐trauma‐informed‐distance‐learning

[ece36915-bib-0127] North, S. (2020). An open letter to the EEB community. Medium.com. Retrieved from https://medium.com/@solonorthrowan/an‐open‐letter‐to‐the‐eeb‐community‐7bd89330e554

[ece36915-bib-0128] O’Brien, L. T. , Bart, H. L. , & Garcia, D. M. (2020). Why are there so few ethnic minorities in ecology and evolutionary biology? Challenges to inclusion and the role of sense of belonging. Social Psychology of Education, 23(2), 449–477. 10.1007/s11218-019-09538-x

[ece36915-bib-0129] Oeberst, A. , von der Beck, I. , Matschke, C. , Ihme, T. A. , & Cress, U. (2019). Collectively biased representations of the past: Ingroup Bias in Wikipedia articles about intergroup conflicts. British Journal of Social Psychology. 10.1111/bjso.12356 31788823

[ece36915-bib-0130] Olimpo, J. T. , & Esparza, D. (2020). Active learning and conceptual understanding in biology In Mintzes J. & Walter E. (Eds.), Active learning in college science (pp. 43–57). Springer.

[ece36915-bib-0131] Orr, A. C. , & Hammig, S. B. (2009). Inclusive postsecondary strategies for teaching students with learning disabilities: A review of the literature. Learning Disability Quarterly, 32(3), 181–196. 10.2307/27740367

[ece36915-bib-0132] Pat‐Horenczyk, R. , Schiff, M. , & Doppelt, O. (2006). Maintaining routine despite ongoing exposure to terrorism: A healthy strategy for adolescents? Journal of Adolescent Health, 39(2), 199–205. 10.1016/j.jadohealth.2005.11.021 16857531

[ece36915-bib-0133] Penner, M. R. (2018). Building an inclusive classroom. Journal of Undergraduate Neuroscience Education, 16(3), A268. 30254542 PMC6153021

[ece36915-bib-0134] Peters, M. A. (2015). Why is My Curriculum White?. Educational Philosophy and Theory, 47(7), 641–646. 10.1080/00131857.2015.1037227

[ece36915-bib-0135] Petersen, C. I. , Baepler, P. , Beitz, A. , Ching, P. , Gorman, K. S. , Neudauer, C. L. , Rozaitis, W. , Walker, J. D. , & Wingert, D. (2020). The Tyranny of Content: “Content Coverage” as a Barrier to Evidence‐Based Teaching Approaches and Ways to Overcome It. *CBE—Life Sciences* . Education, 19(2), ar17 10.1187/cbe.19-04-0079 PMC8697669 32412836

[ece36915-bib-0136] Prince, M. (2004). Does active learning work? A review of the research. Journal of Engineering Education, 93(3), 223–231. 10.1002/j.2168-9830.2004.tb00809.x

[ece36915-bib-0137] Prud’homme‐Généreux, A. (2016a). Assembling a case study tool kit: 10 tools for teaching with cases. Education, 20(2), 37–45.

[ece36915-bib-0138] Prud’homme‐Généreux, A. (2016b). Student‐produced videos for the flipped classroom. Journal of College Science Teaching, 45(3), 58.

[ece36915-bib-0139] Prud’homme‐Généreux, A. (2017). Formulating questions that address student misconceptions in a case study. Journal of College Science Teaching, 46(4), 54.

[ece36915-bib-0140] Prud’homme‐Généreux, A. , Gibson, J. P. , & Csikari, M. (2019). Creating a video case study. Journal of College Science Teaching, 48(4), 46–53.

[ece36915-bib-0141] Prud’homme‐Généreux, A. , Schiller, N. A. , Wild, J. H. , & Herreid, C. F. (2017). Guidelines for producing videos to accompany flipped cases. Journal of College Science Teaching, 46(5), 40.

[ece36915-bib-0142] Ranjan, P. , Kumari, A. , & Chakrawarty, A. (2015). How can doctors improve their communication skills? Journal of Clinical and Diagnostic Research: JCDR, 9(3), JE01 10.7860/JCDR/2015/12072.5712 PMC4413084 25954636

[ece36915-bib-0143] Roach, T. (2014). Student perceptions toward flipped learning: New methods to increase interaction and active learning in economics. International Review of Economics Education, 17, 74–84. 10.1016/j.iree.2014.08.003

[ece36915-bib-0144] Rozek, C. S. , Ramirez, G. , Fine, R. D. , & Beilock, S. L. (2019). Reducing socioeconomic disparities in the STEM pipeline through student emotion regulation. Proceedings of the National Academy of Sciences of the United States of America, 116(5), 1553–1558. 10.1073/pnas.1808589116 30642965 PMC6358706

[ece36915-bib-0145] Saini, A. (2020). Want to do better science? Admit you're not objective. Nature, 579(7798), 175. 32152605 10.1038/d41586-020-00669-2

[ece36915-bib-0146] Sarvary, M. A. , & Gifford, K. M. (2016). Engaging students in large classrooms: turning classical lectures into dialogues using digital pedagogy. Examples, Benefits and Pitfalls. Proceedings of the 8th Annual International Conference on Education and New Learning Technologies (EduLearn16), 7089–7097.

[ece36915-bib-0147] Sawyer, J. E. , Obeid, R. , Bublitz, D. , Schwartz, A. M. , Brooks, P. J. , & Richmond, A. S. (2017). Which forms of active learning are most effective: Cooperative learning, writing‐to‐learn, multimedia instruction, or some combination? Scholarship of Teaching and Learning in Psychology, 3(4), 257 10.1037/stl0000095

[ece36915-bib-0148] Shekhar, P. , Borrego, M. , DeMonbrun, M. , Finelli, C. , Crockett, C. , & Nguyen, K. (2020). Negative student response to active learning in STEM classrooms: A systematic review of underlying reasons. Journal of College Science Teaching, 49(6), 45–54.

[ece36915-bib-0149] Shortlidge, E. E. , Bangera, G. , & Brownell, S. E. (2016). Faculty perspectives on developing and teaching course‐based undergraduate research experiences. BioScience, 66(1), 54–62. 10.1093/biosci/biv167

[ece36915-bib-0150] Shortlidge, E. E. , Bangera, G. , & Brownell, S. E. (2017). Each to their own CURE: Faculty who teach course‐based undergraduate research experiences report why you too should teach a CURE †. Journal of Microbiology & Biology Education, 18(2), 18.2.29 10.1128/jmbe.v18i2.1260 PMC5440172 28656071

[ece36915-bib-0151] Silverthorn, D. U. (2006). Teaching and learning in the interactive classroom. Advances in Physiology Education, 30(4), 135–140. 10.1152/advan.00087.2006 17108239

[ece36915-bib-0152] Silverthorn, D. U. , Thorn, P. M. , & Svinicki, M. D. (2006). It’s difficult to change the way we teach: Lessons from the Integrative Themes in Physiology curriculum module project. Advances in Physiology Education, 30(4), 204–214. 10.1152/advan.00064.2006 17108248

[ece36915-bib-0153] Sisk, V. F. , Burgoyne, A. P. , Sun, J. , Butler, J. L. , & Macnamara, B. N. (2018). To what extent and under which circumstances are growth mind‐sets important to academic achievement? Two meta‐analyses. Psychological Science, 29(4), 549–571. 10.1177/0956797617739704 29505339

[ece36915-bib-0154] Smedley, A. , & Smedley, B. D. (2005). Race as biology is fiction, racism as a social problem is real: Anthropological and historical perspectives on the social construction of race. American Psychologist, 60(1), 16 10.1037/0003-066X.60.1.16 15641918

[ece36915-bib-0155] Smith, K. A. , Douglas, T. C. , & Cox, M. F. (2009). Supportive teaching and learning strategies in STEM education. New Directions for Teaching and Learning, 2009(117), 19–32.

[ece36915-bib-0156] Smith, M. U. , & Scharmann, L. C. (1999). Defining versus describing the nature of science: A pragmatic analysis for classroom teachers and science educators. Science Education, 83(4), 493–509. 10.1002/(SICI)1098-237X(199907)83:4<493:AID-SCE6>3.0.CO;2-U

[ece36915-bib-0157] Steele, C. M. (1997). A threat in the air: How stereotypes shape intellectual identity and performance. American Psychologist, 52(6), 613 10.1037/0003-066X.52.6.613 9174398

[ece36915-bib-0158] Steele, C. M. , & Aronson, J. (1995). Stereotype threat and the intellectual test performance of African Americans. Journal of Personality and Social Psychology, 69(5), 797 10.1037/0022-3514.69.5.797 7473032

[ece36915-bib-0159] Summers, M. M. , Couch, B. A. , Knight, J. K. , Brownell, S. E. , Crowe, A. J. , Semsar, K. , Wright, C. D. , & Smith, M. K. (2018). EcoEvo‐MAPS: An ecology and evolution assessment for introductory through advanced undergraduates. Cbe—life Sciences Education, 17(2), ar18 10.1187/cbe.17-02-0037 29749852 PMC5998322

[ece36915-bib-0160] Sydow, M. , Baraniak, K. , & Teisseyre, P. (2017). Diversity of editors and teams versus quality of cooperative work: Experiments on wikipedia. Journal of Intelligent Information Systems, 48(3), 601–632. 10.1007/s10844-016-0428-1

[ece36915-bib-0161] Tanner, K. D. (2013). Structure matters: Twenty‐one teaching strategies to promote student engagement and cultivate classroom equity. CBE—Life Sciences Education, 12(3), 322–331. 10.1187/cbe.13-06-0115 24006379 PMC3762997

[ece36915-bib-0162] Tanner, K. , & Allen, D. (2007). Cultural competence in the college biology classroom. Cbe—life Sciences Education, 6(4), 251–258. 10.1187/cbe.07-09-0086 18056292 PMC2104499

[ece36915-bib-0163] Tavits, M. , & Pérez, E. O. (2019). Language influences mass opinion toward gender and LGBT equality. Proceedings of the National Academy of Sciences of the United States of America, 116(34), 16781–16786. 10.1073/pnas.1908156116 31383757 PMC6708358

[ece36915-bib-0164] Taylor, A. (2011). Top 10 reasons students dislike working in small groups… and why i do it anyway. Biochemistry and Molecular Biology Education, 39(3), 219–220. 10.1002/bmb.20511

[ece36915-bib-0165] Teaching Tolerance Staff . (2020). A trauma‐informed approach to teaching through coronavirus. Retrieved from https://www.tolerance.org/magazine/a‐trauma‐informed‐approach‐to‐teaching‐through‐coronavirus

[ece36915-bib-0166] Tharayil, S. , Borrego, M. , Prince, M. , Nguyen, K. A. , Shekhar, P. , Finelli, C. J. , & Waters, C. (2018). Strategies to mitigate student resistance to active learning. International Journal of STEM Education, 5(1), 7 10.1186/s40594-018-0102-y 30631697 PMC6310406

[ece36915-bib-0167] Theobald, E. J. , Hill, M. J. , Tran, E. , Agrawal, S. , Arroyo, E. N. , Behling, S. , Chambwe, N. , Cintrón, D. L. , Cooper, J. D. , Dunster, G. , Grummer, J. A. , Hennessey, K. , Hsiao, J. , Iranon, N. , Jones, L. , Jordt, H. , Keller, M. , Lacey, M. E. , Littlefield, C. E. , … Freeman, S. (2020). Active learning narrows achievement gaps for underrepresented students in undergraduate science, technology, engineering, and math. Proceedings of the National Academy of Sciences of the United States of America, 117(12), 6476–6483. 10.1073/pnas.1916903117 32152114 PMC7104254

[ece36915-bib-0168] Tobin, T. J. , & Behling, K. T. (2018). Reach everyone, teach everyone: Universal design for learning in higher education. West Virginia University Press https://wvupressonline.com/node/757

[ece36915-bib-0169] Tseng, M. , El‐Sabaawi, R. W. , Kantar, M. B. , Pantel, J. H. , Srivastava, D. S. , & Ware, J. L. (2020). Strategies and support for Black, Indigenous, and people of colour in ecology and evolutionary biology. Nature Ecology & Evolution, 4, 1288–1290. 32636497 10.1038/s41559-020-1252-0

[ece36915-bib-0170] Vakil, S. , & Ayers, R. (2019). The racial politics of STEM education in the USA: Interrogations and explorations. Taylor & Francis.

[ece36915-bib-0171] van Alten, D. C. D. , Phielix, C. , Janssen, J. , & Kester, L. (2019). Effects of flipping the classroom on learning outcomes and satisfaction: A meta‐analysis. Educational Research Review, 28, 100281 10.1016/j.edurev.2019.05.003

[ece36915-bib-0172] Wagner, C. , Garcia, D. , Jadidi, M. , & Strohmaier, M. (2015). It’s a man’s Wikipedia? Assessing gender inequality in an online Encyclopedia. ICWSM, 454–463.

[ece36915-bib-0173] Wallace, S. L. , Lewis, A. K. , & Allen, M. D. (2019). The state of the literature on student evaluations of teaching and an exploratory analysis of written comments: Who benefits most? College Teaching, 67(1), 1–14. 10.1080/87567555.2018.1483317

[ece36915-bib-0174] Wanelik, K. M. , Griffin, J. S. , Head, M. L. , Ingleby, F. C. , & Lewis, Z. (2020). Breaking barriers? Ethnicity and socioeconomic background impact on early career progression in the fields of ecology and evolution. Ecology and Evolution, 10(14), 6870–6880. 10.1002/ece3.6423 32760497 PMC7391347

[ece36915-bib-0175] Wheeler, L. B. , Mulvey, B. K. , Maeng, J. L. , Librea‐Carden, M. R. , & Bell, R. L. (2019). Teaching the teacher: Exploring STEM graduate students’ nature of science conceptions in a teaching methods course. International Journal of Science Education, 41(14), 1905–1925. 10.1080/09500693.2019.1647473

[ece36915-bib-0176] White, P. J. T. , Heidemann, M. , Loh, M. , & Smith, J. J. (2013). Integrative cases for teaching evolution. Evolution: Education and Outreach, 6(1), 17.

[ece36915-bib-0177] Winberg, C. , Adendorff, H. , Bozalek, V. , Conana, H. , Pallitt, N. , Wolff, K. , Olsson, T. , & Roxå, T. (2019). Learning to teach STEM disciplines in higher education: A critical review of the literature. Teaching in Higher Education, 24(8), 930–947. 10.1080/13562517.2018.1517735

[ece36915-bib-0178] Winkelmes, M.‐A. , Bernacki, M. , Butler, J. , Zochowski, M. , Golanics, J. , & Weavil, K. H. (2016). A teaching intervention that increases underserved college students’ success. Peer Review, 18(1/2), 31–36.

[ece36915-bib-0179] Wood, S. , Henning, J. A. , Chen, L. , McKibben, T. , Smith, M. L. , Weber, M. , Zemenick, A. , & Ballen, C. J. (2020). A scientist like me: Demographic analysis of biology textbooks reveals both progress and long‐term lags. Proceedings of the Royal Society B, 287(1929), 20200877 10.1098/rspb.2020.0877 32576104 PMC7329037

[ece36915-bib-0180] Woodley, X. , Hernandez, C. , Parra, J. , & Negash, B. (2017). Celebrating difference: Best practices in culturally responsive teaching online. TechTrends, 61(5), 470–478. 10.1007/s11528-017-0207-z

[ece36915-bib-0181] Wright, A. M. , Schwartz, R. S. , Oaks, J. R. , Newman, C. E. , & Flanagan, S. P. (2019). The why, when, and how of computing in biology classrooms. F1000Research, 8, 1854 10.12688/f1000research.20873.1 32025290 PMC6971840

[ece36915-bib-0182] Xing, J. , & Vetter, M. (2020). Editing for equity: Understanding instructor motivations for integrating cross‐disciplinary Wikipedia assignments. First Monday, 25(6). 10.5210/fm.v25i6.10575

[ece36915-bib-0183] Xu, D. , & Jaggars, S. S. (2014). Performance gaps between online and face‐to‐face courses: Differences across types of students and academic subject areas. The Journal of Higher Education, 85(5), 633–659. 10.1353/jhe.2014.0028

[ece36915-bib-0184] Zhao, N. , Wardeska, J. G. , McGuire, S. Y. , & Cook, E. (2014). Metacognition: An effective tool to promote success in college science learning. Journal of College Science Teaching, 43(4), 48–54. 10.2505/4/jcst14_043_04_48

[ece36915-bib-0185] Zuberi, T. , & Bonilla‐Silva, E. (2008). White logic, white methods: Racism and methodology. Rowman & Littlefield Publishers https://rowman.com/isbn/9780742542808/white‐logic‐white‐methods‐racism‐and‐methodology

[ece36915-bib-0186] Zydney, J. M. , Warner, Z. , & Angelone, L. (2020). Learning through experience: Using design based research to redesign protocols for blended synchronous learning environments. Computers & Education, 143, 103678 10.1016/j.compedu.2019.103678

