# Supplementary material for: From panic to pedagogy: Using online active learning to promote inclusive instruction in ecology and evolutionary biology courses and beyond
Source: Ecol Evol. 2020 Oct 29;10(22):12581–612. doi: 10.1002/ece3.6915 (PMC7679552; doi:10.1002/ece3.6915)
Supplement: Supplementary file 1 — Appendix S1 [file ECE3-10-12581-s001.docx]

**Introduction to Active Learning Class Activity**

*Note: This activity is meant to be modified by individual instructors for use in their courses, but please credit this paper as the original source. Please see the blue text for areas that should be edited to fit individual course needs.*

*This case was originally used in a face-to-face session for a 300-student, pre-nursing anatomy and physiology course to prepare students for weekly group work and case studies. In this anatomy and physiology course there are two lecture meetings per week. One of those periods is spent in a semi-interactive lecture and the other is entirely active where students work together to solve case studies relevant to that week’s content. Students work in groups of four and are aided by undergraduate teaching assistants (UTAs, students who previously took and did well in the course) and the instructor. The UTAs take a separate pedagogy course, taught by the same instructor. In addition to mentoring, UTAs also develop their own case studies and some of those cases have been published at the National Center for Case Study Teaching in Science (for more, see cases authored by Harris in the database;* <https://sciencecases.lib.buffalo.edu/teaching/directory/authors.html?keyword=harris&submit=Search>).

*This activity was conducted using an interrupted format (i.e., parts of the case were released on the learning management system over the class period). But, this activity, or parts of it, could be incorporated into online group work, an assignment, or a discussion forum.*

Purpose: [*instructors – please edit this part to reflect specifics of your course*]. Today’s activity is a practice case study over the concept of and research on active learning. The purpose of this activity is to: 1) get you acquainted with your groupmates, 2) give you the opportunity to practice working together, 3) mimic the class set-up for additional case studies, and 4) to learn about the benefits of active learning in the classroom.

Instructions: [*instructors – please edit this part to reflect specifics of your course*]. Please get into your groups and work with your groupmates to discuss and answer the below questions. Write your answers on one piece of paper for the whole group, there is no need to re-write the question, just put the question number and your answer. Please do not split up questions among group members, everyone should be involved in answering each question. The concepts covered in class activities build across the assignment, if groups split up the work, students miss out on important information. Additionally, a major goal of this class is to practice working as a team. Many of you are in pre-health or allied health programs. Teamwork is critical in healthcare and thus I want you all to practice working together, to consider multiple viewpoints, and to learn from and teach your peers. Answers will be turned in at the end of the class period.

Grading: [*instructors – please edit this part to reflect specifics of your course*]. Today’s case study will not be graded. Future case study assignments will be graded via Top Hat concept checks throughout the class period, please see the syllabus for more details. You will go over the answers to this case study in next week’s discussion section.

Tools: [*instructors – please edit this part to reflect specifics of your course*]. For today’s activity you will need at least one laptop computer or WiFi-enabled device for the group. Please write your answers on notebook paper, there is no need to re-write the question, just provide the number and the answer. You do not need to use complete sentences, bullets, lists, or pictures are okay, however, if a question asks for a specific format of answer (e.g., picture), please provide that.

Learning Goals: [*instructors – please edit this part to reflect specifics that pertain to your use of the course*].

After completion of this case, students will have, will know, or will be able to:

- Compare and contrast learning and memorizing
- Compare and contrast the role of students and professors in making a course “good”
- Explain what grades represent and what is means to earn an “A”
- Define active learning and learning outcomes
- Locate primary research articles using Google Scholar
- Describe the basics of a research experiment and the parts of a scientific paper
- Discuss the empirical data related to active learning in the classroom
- Practiced metacognitive thinking about the courses in which they are enrolled

********************************************************************************

**Group Introductions:**

Before we start – let’s get to know each other a bit. Once you’re in your groups, please introduce yourselves, make sure to tell everyone the following: your name, your major, your career plans, and something boring about you.

Once you’ve all been introduced, find one thing that all of you have in common (the more random, the better), try to get something that you think no other group will also have in common. E.g., we all play a musical instrument; we all have blue backpacks, etc. (for example, we all go to this university is not a very good common item).

Once you’ve done this, I will open a Top Hat question and ask what you all had in common. Pick one person to answer and then type your groupmates first names and then your thing. (e.g., Juan, Devin, Gloria, Shu – we all have been to Albuquerque, NM). These answers will not be graded, I just want to practice using the technology.

**Part I – What is the point of this course?**

Most of you said you were taking this course to either 1) get an A, or 2) because it was required. Let’s build on both of those ideas *[I had previously polled the students to see why they were taking my course - you may need to remove this part, or add a question of this nature to the list]*. Read each question and think about an answer individually, then come together as a group to discuss each one. Write a short summary of your group’s answer on your collective paper or Google doc [modify instructions as necessary]. Please take turns writing and make sure everyone has the opportunity to contribute to the group discussion.

1. How do you think the information in this course will apply to your life and/or career plans?
2. What does an “A” on a transcript symbolize to a future program or employer?
3. What is a good course? What makes a course good by definition?
4. What is the role of a college professor?
5. What is the role of a college student?
6. Think about the role of students and of professors in creating a “good” course. Discuss with your group and then complete a version of the below table (put at least 3 things in each box).

|  | Student(s) | Professor(s) |
| --- | --- | --- |
| A good (student/professor) does/is… |  |  |
| A not so good (student/professor) does/is… |  |  |

**Part II – What is Active Learning**

1. Discuss with your group. What does it mean to truly learn something? Is it the same or different from memorizing something? After you discuss, jot down a quick summary of your discussion.
2. What is active learning? Find a definition online and cite your source.
3. What are learning outcomes? [Use page 5-9 of the following document for your answer; instructors may want to insert their university handbook here] <https://www.depts.ttu.edu/opa/resources/docs/Writing_Learning_Outcomes_Handbook3.pdf>
4. How does one measure learning? Discuss with your group and then see [Page 12 of the above link].

**Part III – Research**

One of the major focuses of large universities, like Texas Tech, is research. The majority of faculty (professors) at TTU, and other research institutions, conduct some form or research or scholarly work. This requirement to produce new knowledge is a major job duty for most of your professors (other duties include teaching and service).

When we say research, here we mean creating new information. Reading and doing library research and literature reviews are certainly important for learning what knowledge exists, but our main goal is to ask questions, develop hypotheses, do experiments/studies, collect data, and generate new information. Some studies are short and can be finished relatively quickly, whereas others can take years. Some studies are expensive to complete, whereas other studies can be done with minimal supplies. Some studies involved the use of humans, animals, plants, microbes, or cells, and others are done without living organisms. All types of studies are important.

Many, but not all, studies are designed to test a specific hypothesis and use the scientific method. Generally, one initially determines what is known and what is still unknown about a specific topic or question, this is done via lots and lots of reading. Next, one proposes a hypothesis and designs an experiment to test that hypothesis. The experiment gets ethical approval (if needed) and then data collection can begin. Once data are collected, they are analyzed, typically with statistics, and one can determine if the hypothesis was supported, or not.

1. What is a hypothesis?
2. What is an independent variable?
3. What is a dependent variable?
4. What is the purpose of a control group in an experiment?
5. What is a confounding variable?
6. What is a covariate?

**Part IV – Dissemination of Research**

[Depending on the level of students or needs of the course structure, instructors may want to edit or remove this section]

You may have heard the phrase “publish or perish”. This pertains to the culture of academia (research at universities). Researchers need to not only generate new knowledge, but they also must disseminate that new information to others (new data does no good locked away in a single lab). The traditional way we do this is via conference presentations and via scientific publications. These publications, or scientific journal articles, have specific formatting requirements and they are rigorously scrutinized and critiqued by our peers prior to being published in an academic journal.

Scientific journal articles are considered primary literature as they are written by the scientist(s) who conducted the experiments and performed the analyses. Scientific articles are also subjected to a rigorous peer-review process. For writing to be effective and worthy of publication it must be clear, succinct, and understandable. Scientific articles (generally) follow the format listed below.

Abstract: This is a stand-alone summary of the research. Abstracts have strict word limits (150-350 words, depending on the journal). General framework for the abstract is one sentence about the background/big picture of the study, one sentence about the question and hypothesis, one to two sentences about the methods of the study, one to two sentences about the results, one sentence about the conclusion and how this study relates to the big picture.

Introduction: This section sets up the rationale for the study and should go from broad to specific. Generally, the introduction has ~5-7 paragraphs, each with a specific point. This section should tell the reader 1) the big picture to put the research into context, 2) a bit on what’s known, 3) what is still unknown, 4) the question/hypothesis this research will address (should be addressing what is unknown from point 3), 5) why this is important. Ideally, by the time the reader is done with the introduction they should be thinking *this research is incredibly important and desperately needed, why has this not been done before?!*

Methods: This is the section where you’ll find what, exactly, the researchers did and how they did it. This section should be detailed enough for someone in that same field to be able to replicate the experiment and it should list a clear overview and/or timeline of the study, what variables were measured and how data were analyzed. The use of subheading is common in this section and this practice makes the paper easier to read. The first subheading of the methods section is always Animals/Subjects/Participants.

Results: This section tells the reader what the authors found. This is where data and statistical results are presented; Figures and Tables also go here. The data are not interpreted in this section. The use of subheadings in this section is also common.

Discussion: This is where the authors interpret their results and discuss whether the data support or fail to support the hypothesis (note, we never prove anything in science). This is also the section where authors will address any limitations or caveats of the study, and will integrate their findings into the big picture (e.g., how do these results fit in within the field? What do they tell us about the hypothesis being tested?).

Conclusion: This section is not always present. It is a nice, big-picture summary of the study, the results and why the results are important.

Acknowledgements: In this section authors will thank various people who helped with the project and the funding sources.

References: This section lists all of the sources specifically referenced in the paper. References should be other primary literature articles, or, occasionally, text or reference books. In the field of biology, each journal has their own formatting preference for references.

No questions to answer for this section.

**Part V- Is Active Learning Helpful? Integrating learning with research.**

When you think of research on a college campus, you may initially think of studies involving cancer, HIV, addiction, or other medically relevant topics. These are certainly important research avenues, but people study a wide range of subjects, including learning and pedagogy. Multiple people investigate how students learn and what forms of teaching practices are the most effective.

We discussed active learning in class [one lecture in this course was devoted to active learning, study methods, and research; please edit as necessary]. The next thing I’d like you all to think about is whether active learning is helpful and how we could empirically determine if active learning benefits students.

1. Brainstorm with your group and then list 1-3 possible research questions pertaining to the impacts or effects of active learning.
2. If needed, refine your question to be more specific, and then develop the below:
   1. A (testable) hypothesis based on your question
   2. Make sure to list your dependent variable (e.g., the outcome(s) you plan to measure) and the independent variable (e.g., your treatment groups or the thing you are manipulating).
   3. A brief experimental design to test your hypothesis
   4. A prediction that would support your hypothesis

*For example (please do not use this exact stuff for your answer):*

*Q: Does active learning help students in intro biology?*

*H: active learning is more effective than passive learning for retaining info in intro bio*

*IV: two groups of intro bio students, taught by the same professor, one class gets active learning and the other class does not. (might want to control for things like GPA, class time of day, and other potential confounding variables).*

*DV: learning as measured by score on a competency test the semester after taking intro bio.*

*Design: the two classes would cover the same material, but the instructor would use an active form (e.g., case studies) for one class and passive learning (e.g., lecture) for the other class.*

*Prediction: students who were in the active learning class will score higher on the competency test compared to students in the passive learning class.*

**Part VI - What do the data say?**

Lots and lots of people have done what you just did in the above section. That is, they do research on teaching and learning practices in a variety of settings.

There are several databases that one can use to find the research articles written by faculty (and students). One of these is Google Scholar (<https://scholar.google.com/>).

In this section, you will use Google Scholar to locate active-learning related research. Copy the title of the article you’d like to locate and paste the title in the Google Scholar search bar. Then, you will look at the research article to determine what the authors did and what they found.

Choose 2 out of the below papers that deal with active learning and/or teamwork in the classroom. For each one, use Google Scholar to locate the article (web or pdf version), and then list:

a) the question or hypothesis the authors addressed,

b) the study population (e.g., A&P students; med students; etc.),

c) intervention or treatment (the independent variable; IV) and the control group,

d) what outcome did they measure (the dependent variable; DV)

e) the main conclusions of the study.

You should be able to get most of this information from the article abstract, but you may need to look at other parts of the paper. If any part of the a-e sections are not applicable to the article you chose, just write NA.

Optional papers are listed below questions on the next page.

1. Paper 1 (title or authors):
   1. Hypothesis:
   2. Study population:
   3. IV:
   4. DV:
   5. Main conclusion:
2. Paper 2 (title or authors):
   1. Hypothesis:
   2. Study population:
   3. IV:
   4. DV:
   5. Main conclusion:

Papers from which you can choose: [instructors may want to add or remove references from this list]

Search in google scholar. You can access the web file or pdf by clicking the link on the right-hand side. You may need to link your Google account to the school library (this can be done by clicking the three bars on the left side, clicking settings, and then clicking library links).


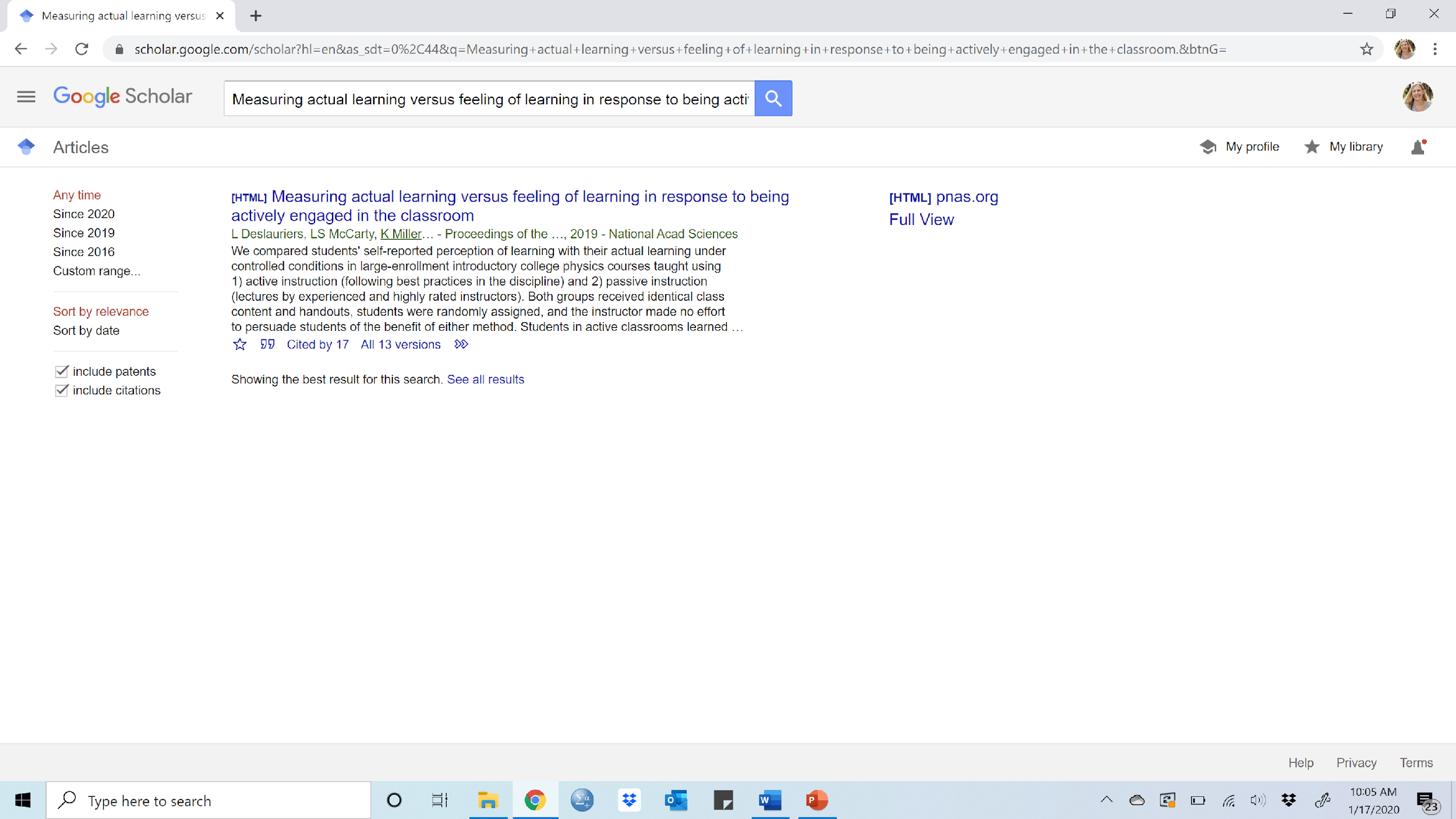


- Armbruster, P., Patel, M., Johnson, E., & Weiss, M. (2009). Active learning and student-centered pedagogy improve student attitudes and performance in introductory biology. *CBE—Life Sciences Education*, *8*(3), 203-213.
- Ballen, C. J., Wieman, C., Salehi, S., Searle, J. B., & Zamudio, K. R. (2017). Enhancing diversity in undergraduate science: Self-efficacy drives performance gains with active learning. *CBE—Life Sciences Education*, *16*(4), ar56.
- Carvalho, H., & West, C. A. (2011). Voluntary participation in an active learning exercise leads to a better understanding of physiology. *Advances in Physiology Education*, *35*(1), 53‐58.
- Deslauriers, L., McCarty, L. S., Miller, K., Callaghan, K., & Kestin, G. (2019). Measuring actual learning versus feeling of learning in response to being actively engaged in the classroom. *Proceedings of the National Academy of Sciences*, *116*(39), 19251-19257.
- Frasier, T. R., & Roderick, C. (2011). Improving how evolution is taught: facilitating a shift from memorization to evolutionary thinking. *Evolution: Education and Outreach*, *4*(2), 298.
- Freeman, S., O'Connor, E., Parks, J. W., Cunningham, M., Hurley, D., Haak, D., ... & Wenderoth, M. P. (2007). Prescribed active learning increases performance in introductory biology. *CBE—Life Sciences Education*, *6*(2), 132-139.
- Freeman, S., Eddy, S. L., McDonough, M., Smith, M. K., Okoroafor, N., Jordt, H., & Wenderoth, M. P. (2014). Active learning increases student performance in science, engineering, and mathematics. *Proceedings of the National Academy of Sciences*, 8410‐8415.
- Goodman, B. E., Barker, M. K., & Cooke, J. E. (2018). Best practices in active and student-centered learning in physiology classes. *Advances in Physiology Education*, 42, 417-423.
- Graham, M. J., Frederick, J., Byars-Winston, A., Hunter, A. B., & Handelsman, J. (2013). Increasing persistence of college students in STEM. *Science,* 341(6153), 1455-1456.
- Haak, D. C., HilleRisLambers, J., Pitre, E., & Freeman, S. (2011). Increased structure and active learning reduce the achievement gap in introductory biology. *Science*, *332*(6034), 1213-1216.
- Lerner, S., Magrane, D., & Friedman, E. (2009). Teaching teamwork in medical education. *Mount Sinai Journal of Medicine: A Journal of Translational and Personalized Medicine*, *76*(4), 318-329.
- McCoy, L., Pettit, R. K., Kellar, C., & Morgan, C. (2018). Tracking active learning in the medical school curriculum: a learning-centered approach. *Journal of medical education and curricular development*, *5*, 2382120518765135.
- Snyder, J. J., Sloane, J. D., Dunk, R. D., & Wiles, J. R. (2016). Peer-led team learning helps minority students succeed. *PLoS biology*, *14*(3), e1002398.
- Theobald, E. J., Hill, M. J., Tran, E., Agrawal, S., Arroyo, E. N., Behling, S., ... & Grummer, J. A. (2020). Active learning narrows achievement gaps for underrepresented students in undergraduate science, technology, engineering, and math. *Proceedings of the National Academy of Sciences*, *117*(12), 6476-6483.
- Weaver, G. C., Russell, C. B., & Wink, D. J. (2008). Inquiry-based and research-based laboratory pedagogies in undergraduate science. *Nature chemical biology*, *4*(10), 577.
